# Human Endometriotic Lesion‐Derived Small Extracellular Vesicles Impair Macrophage Function in the Peritoneal Microenvironment

**DOI:** 10.1002/jev2.70227

**Published:** 2026-02-19

**Authors:** Yifan Wang, Zhixing Jin, Abigail Freeman Blatchford, Banayot Hosh, Malak Amer, Ayazhan Akhatova, Krina Zondervan, Erin Greaves, Rebecca Dragovic, Christian M Becker, Jen Southcombe

**Affiliations:** ^1^ Oxford Endometriosis Care Centre, Nuffield Department of Women's & Reproductive Health University of Oxford Oxford UK; ^2^ Department of Obstetrics and Gynecology The First Affiliated Hospital of Soochow University Suzhou Jiangsu Province People's Republic of China; ^3^ Division of Biomedical Sciences, Warwick Medical School University of Warwick Coventry UK

**Keywords:** endometrial epithelial organoid, endometriosis, peritoneal fluid, phagocytosis, small extracellular vesicles

## Abstract

Endometriosis (EM) is a chronic inflammatory disease that affects ∼10% of women during reproductive age. It is characterised by ectopic (ECT) growth of endometrial‐like tissue mainly in the pelvic cavity. Small extracellular vesicles (sEVs) mediate cellular interactions, but their function remains poorly understood in the pathogenesis of EM. 3D endometrial epithelial organoids (EEOs) from ECT lesions and eutopic (EUT) endometrium from EM patients and controls were established to investigate sEVs. Multiplex bead‐based flow cytometry revealed CD133/1 and EpCAM as dominant markers on EEO‐sEVs, with ECT EEO‐sEVs showing upregulation of CD44, CD29 and downregulation of EpCAM compared to EUT EEO‐sEVs. Peritoneal fluid (PF)‐sEVs displayed high and correlated CD133/1 and EpCAM expression, indicating a major contribution from endometrial epithelial (EE) cells, alongside sEVs of lymphocyte and endothelial origin. Functionally, both ECT EEO‐sEVs and PF‐sEVs from EM patients significantly suppressed macrophage phagocytosis, as assessed by pH‐sensitive fluorescent bioparticles. The effect was reversed by CD47 blockade. The coexpression of CD47 with CD133/1 and EpCAM on PF‐sEVs indicates the involvement of EE cell‐derived sEVs in CD47/SIRP‐α mediated suppression. This study provides the first thorough characterisation of EE‐derived sEVs utilising EEO models in EM and demonstrates their potential immunomodulatory role in the peritoneal microenvironment via CD47/SIRP‐α signalling.

## Graphical abstract

sEVs in the peritoneal microenvironment originate from immune cells and endometrium. In endometriosis (EM), lesion‐derived sEVs show upregulation of CD44 and CD29 and downregulation of EpCAM compared to those from eutopic endometrium. This contributes to EM specific sEV signature in the peritoneal cavity and likely suppresses macrophage phagocytosis via the CD47/SIRP‐α pathway.

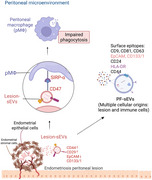

## 1 Introduction

Endometriosis (EM) is a chronic inflammatory disorder characterised by the presence of endometrial‐like tissue outside the uterine cavity, primarily on the pelvic peritoneum and ovaries (Zondervan et al. 2020). It affects approximately 10% of women of reproductive age and is associated with a wide range of symptoms, including chronic pelvic pain, dysmenorrhea, dyspareunia, infertility and fatigue (Burney and Giudice 2012). Despite its high prevalence and significant impact on women's quality of life, it takes an average of 7–9 years to receive a diagnosis (Nnoaham et al. 2011), leading to a substantial economic burden (Simoens et al. 2012).

EM lesions are heterogeneous, but typically comprise cells from stroma, epithelium and endothelium, with infiltrating immune cells, blood vessels and nerves (Zondervan et al. 2020). EM is frequently classified into four stages (I–IV) based on the revised American Society for Reproductive Medicine (rASRM) scoring system, which considers the size, location and depth of endometriotic lesions, as well as the presence of adhesions (Canis et al. 1997). However, there is poor correlation between the stage of EM and the severity of symptoms experienced by patients (Vercellini et al. 2007). Additionally, current treatments, including hormonal therapies and surgical interventions, are not curative and are often associated with high recurrence rates and resistant pain (Becker et al. 2017). This discrepancy between disease stage and symptom severity, combined with limited effective treatments, highlights the importance of understanding the mechanisms underlying the disease. As a result, this will enable the development of targeted, personalised therapeutic approaches.

The most widely accepted theory of how endometrial‐like tissue is present in the pelvic cavity is Sampson's theory of retrograde menstruation, which proposes that during menstruation, endometrial fragments are refluxed through the fallopian tubes into the peritoneal cavity, where they implant and grow (Sampson 1927). However, retrograde menstruation occurs in most women physiologically, and additional factors are believed to contribute to the establishment and progression of endometriotic lesions (Halme et al. 1984). Current theories suggest that EM is a multifactorial disorder, involving a complex interplay between hormonal, immunological and genetic factors (Zondervan et al. 2018). The peritoneal microenvironment in EM is characterised by an altered immune cell composition and function, with increased macrophage recruitment and activated macrophages with impaired phagocytic activity, reduced natural killer cell cytotoxicity, and an imbalance between pro‐ and antiinflammatory cytokines (Izumi et al. 2018). These immune alterations are thought to contribute to the survival and growth of ectopic (ECT) endometrial tissue (lesions) and the development of EM‐associated pain (Burney and Giudice 2012).

In recent years, the role of extracellular vesicles (EVs) in intercellular communication and disease pathogenesis has gained significant attention (Yáñez‐Mó et al. 2015). Among the different types of EVs, small extracellular vesicles (sEVs) have emerged as key players in various physiological and pathological processes, including cancer (Azmi et al. 2013), neurodegenerative diseases (Howitt and Hill 2016) and reproductive disorders (Salomon and Rice 2017).

Endometrial stromal (ES) cells constitute the most abundant cell type in both the eutopic (EUT) endometrium (Lv et al. 2022; Marečková et al. 2024) and ECT endometrium (Fonseca et al. 2023), making them a primary focus of sEVs research in EM. An RNA sequencing study has revealed that the RNA profiles of ES cell‐derived EVs from ovarian endometrioma differ significantly from those of EUT endometrium in patients without EM (Wu et al. 2020). These ES cell‐derived sEVs have been shown to promote disease progression by modulating the activities of both stromal and nonstromal cells within the peritoneal microenvironment. For example, studies have found that the ECT ES cell‐derived sEVs deliver actin filament‐associated protein 1‐antisense RNA 1 (AFAP1‐AS1) to ECT ES cells to promote cell migration and invasion (Wang et al. 2022). Beyond autocrine signalling, Qiu et al. (2019) revealed that sEVs from ES cells of EM patients exhibit differential profiles of lncRNA antisense hypoxia inducible Factor 1 alpha (HIF‐1α) that promote proangiogenic properties in endothelial cells. The lncRNA HIF‐1α derived from ES cells targets VEGF, a potent proangiogenic molecule highly expressed in EM lesions and PF of EM patients (Qiu et al. 2019). In terms of immune modulation, sEV derived from EUT ES cells induces macrophage polarisation into an antiinflammatory subtype with decreased phagocytotic abilities, leading to increased lesion size in mice (Sun et al. 2018). Furthermore, ECT ES cells from patients with recurrent ovarian EM carrying pseudogene LGMNP1 induce antiinflammatory polarisation of macrophages via the secretion of sEV (Sun et al. 2022). sEV‐mediated bidirectional crosstalk between peritoneal macrophage (pMΦ) and endometrial epithelial (EE) cells has been observed (Wang et al. 2023). For example, pMΦ‐sEV can transfer miR‐22‐3p to ES cells, enhancing cell proliferation, migration and invasion through the regulation of the SIRT1/NF‐κB signalling pathway (Zhang et al. 2020).

Despite the growing interest in ES cell‐derived sEVs, relatively little is known about the role of sEVs from EE cells in EM. EE cells harbour more oncogene mutations than ES cells (Suda et al. 2019), and recent advances in spatial transcriptomics of endometriotic lesions have revealed that the endometrial epithelium plays a crucial role in modulating the local immune environment, particularly in relation to macrophages (Burns et al. 2025). To date, only one major study has specifically investigated EVs from EE cells. Zhang et al. (2022) demonstrated that EUT EE cell‐derived EVs carry miR‐30c that supress invasion and migration of ECT EE cells.

These cell‐specific sEV alterations in EM are reflected in the peritoneal microenvironment. Several studies have demonstrated altered miRNA profiles in sEVs isolated from the peritoneal fluid (PF) of EM patients (Lee et al. 2021; Chen et al. 2019; Nazri et al. 2023). A previous proteomic study from our group reported the presence of sEVs in the PF of EM patients with distinct protein profiles compared to controls (Nazri et al. 2020).

Although most characterisation and functional studies to date have focused on the RNA content of sEVs, surface epitopes still remain underexplored in PF‐sEVs and particularly those of EE cell origin from ECT endometrium. Surface proteins can regulate recipient cell activities directly, including through antigen presentation without requiring internalisation (Mallegol et al. 2007). Moreover, surface markers are often indicative of the cellular origin of sEVs (Villarroya‐Beltri et al. 2014), serving as molecular ‘fingerprints’ of their parent cells and reflecting the complexity of the surrounding microenvironment.

To address these gaps, our study aims to establish 3D EE organoid (EEO) models to investigate the marker expression profile of EE cell‐derived sEVs from ECT and EUT endometrium. We further aim to explore the heterogeneity and cellular origins of these sEVs within the peritoneal microenvironment of EM. Finally, we examined their role in modulating macrophage phagocytic activity.

## 2 Methods

### 2.1 Patient Recruitments and Sample Collection

Biological samples and clinical data were collected as part of the prospective FENOX (17/SC/0664) and ENDOX (09/H0604/58) studies from women undergoing planned laparoscopic surgery for suspected EM (Tapmeier et al. 2020). Samples and data were collected, processed and stored according to World Endometriosis Research Foundation Endometriosis Phenome and Biobanking Harmonisation Project (WERF EPHect) protocols (Becker et al. 2014; Fassbender et al. 2014; Rahmioglu et al. 2014; Vitonis et al. 2014). Hormonal use was self‐reported, and menstrual cycle phase was self‐reported or confirmed by histology of endometrial biopsy samples taken at the time of laparoscopy. EM was staged according to the rASRM classification (Canis et al. 1997). Control patients (CONs) were defined as those patients undergoing the laparoscopy with no visible EM lesions, matched for age and menstrual cycle phase.

Patient exclusion criteria included malignancy, pregnancy or breastfeeding for at least 3 months at the time of surgery. All PF samples were pure, with no lavage and minimal blood contamination. EUT endometrium samples were obtained using a disposable endometrial sampler (Wallach Endocell, CooperSurgical, USA), and ECT endometrium (lesions) were collected during surgery. EUT or ECT endometrium was either stored in CS10 solution (STEMCELL Technologies, Canada) at −80°C or processed for immediate culture.

### 2.2 EEO Establishment

ECT endometrium samples were processed fresh and enzymatically digested in prewarmed advanced DMEM/F‐12 (Thermo Fisher Scientific, USA) containing 1 mg/mL Collagenase V (Sigma–Aldrich, USA) for 30–60 min on a rotator (16 rpm) at 37°C. EUT endometrium samples, either fresh or previously frozen, were thawed if necessary before undergoing the same enzymatic digestion protocol. Following digestion, EUT endometrium samples were passed sequentially through a 100 and 40 µm disposable strainer (Corning, USA). The 40 µm strainer retained EE gland‐like structures, while the ES cells, presents single cells, passed through. To recover the retained EE cells, the 40 µm strainer was rinsed with 50 mL of advanced DMEM/F12 media. For ECT endometrium, the digested tissue was passed through a 70 µm strainer (Corning) and the filtrate collected in a 50 mL tube. EE cells were pelleted by centrifugation (500 × *g*, 5 min) and resuspend in 250 µL Matrigel (Corning) then droplets were deposited in 48‐well plates (20 µL per well). EE organoids (EEOs) were cultured in 250 µL expansion media (ExM) as previously described (Table S1). ROCK inhibitor Y27632 (10 µM, Abcam, UK) was added in ExM for the first 3 days of each passage. ExM was changed every 2–3 days.

Matrigel‐embedded EEOs were passaged every 7–10 days. EEOs were mechanically scraped into small pieces and centrifuged (600 × *g*, 6 min). The supernatant was discarded and EEO were resuspended in 150 µL advanced DMEM/F12 media and dissociated by pipetting 300 times. Samples were centrifuged (600 × *g*, 6 min) and further dissociated by pipetting 80 times. Dissociated EEOs were resuspended in Martrigel and plated as 20 µL droplets in 48‐well plates. Established EEOs were amplified, cryopreserved (P0–P2) and subjected to sEV collection. Unless otherwise stated, EEOs of low passage number (P3–P5) were used for the experiments described.

### 2.3 Immunofluorescence Analysis of EEO Structure

EEOs were prepared for antibody staining by culturing for 7–12 days on an 8‐well microscopy chamber slide (ibidi, Thermo Fisher Scientific). Once ready, whole‐mount staining was performed on EEO within Matrigel. Briefly, EEOs were washed with PBS and fixed for 30 min using 4% formaldehyde aqueous solution (VWR, USA) at room temperature, followed by permeabilisation using 0.5% Triton X‐100 (Sigma–Aldrich) in PBS for 30 min. After blocking with 5% BSA/0.2% Triton X‐100/0.05% Tween‐20 (Sigma–Aldrich) in PBS for 2–3 h, the EEOs were incubated with unconjugated primary antibodies (EpCAM) or conjugated antibodies (E‐cadherin, pan‐cytokeratin) overnight. The following day, EEOs were washed three times with PBS and incubated with appropriate secondary antibodies for 2–3 h at room temperature. EEOs were then mounted using mounting medium with DAPI (Abcam). Zstack images were acquired using a Leica TCS SP8 confocal laser scanning microscope. Details of all antibodies used are listed in Table S2.

### 2.4 pMΦ Culture

pMΦ were isolated from fresh PF by a previously described method (Wu et al. 2002). In brief, peritoneal cells were collected by Ficoll (Cytiva, USA) centrifugation (800 × *g*, 20 min), and the mononuclear cell layer was collected and washed with RPMI‐1640 media (Thermo Fisher Scientific) at 300 × *g* for 10 min. The cells were counted and seeded in 24‐well plates for 30 min for adherence. The adherent cells were maintained in complete media composed of RPMI‐1640 media (Thermo Fisher Scientific) with 100 U/mL penicillin/streptomycin (P/S) (Sigma–Aldrich) and 10% foetal calf serum (FCS) (Sigma–Aldrich). Culture media was changed every 2–3 days to maintain optimal growth conditions. For sEV isolation, cells were cultured in EV‐depleted medium (containing FCS pre‐ultracentrifuged at 150,000 × *g* for 16 h at 4°C) from Days 5 to 7. The resulting conditioned media (CM) was collected after 48 h for subsequent sEV isolation.

### 2.5 sEV Isolation

For sEV collection, cryopreserved EEOs were thawed and cultured for one passage to allow recovery and then dissociated into single cells during the second passage. EEOs were recovered by liquifying the Matrigel drop with cell recovery solution (Corning) for 50 min at 4°C. Subsequently, the EEOs were dissociated using TrypLE (Thermo Fisher Scientific) on a rotator for 30 min; the mixture was filtered through a 40 µm cell strainer and centrifuged at 300 × *g* for 5 min. Cells were resuspended in Matrigel at a density of 500–1000 cells/µL. Between Days 4 and 7 or 7 and 10, EEOs were cultured in EV‐depleted ExM (pre‐ultracentrifuged at 150,000 × *g*, 4 h, at 4°C), and 72 h CMs were collected for sEV isolation.

sEVs were isolated from PF samples that had been thawed at 37°C, as well as from the CM of pMΦ and EEO. All samples were centrifuged at 1500 × *g* for 10 min, and the supernatant retained, followed by centrifugation at 16,000 × *g* for 30 min. For PF samples, 0.5–1 mL of supernatant was run through qEV original/35 nm size exclusion chromatography (SEC) columns (IZON Science), according to the manufacturer's instructions. Fractions 6–9 were pooled and filtered using a 0.22 µm membrane (Corning). sEVs were concentrated by ultracentrifugation at 150,000 × *g* for 2 h at 4°C and resuspended in PBS or RIPA lysis buffer. The supernatant from the CM of pMΦ and EEO (from 22 wells of 48‐well plates) was ultracentrifuged at 150,000 × *g* for 2 h, and pellets were resuspended in 500 µL of PBS. These vesicles were run through SEC columns, and Fractions 6–9 were collected, filtered (0.22 µm membrane) and concentrated by ultracentrifugation at 150,000 × *g* for 2 h at 4°C and resuspended in either PBS or RIPA lysis buffer. sEVs were characterised based on the MISEV 2023 guidelines (Welsh et al. 2024).

For all ultracentrifugation steps, samples were placed in Ultra‐Clear ultracentrifuge tubes (5 mL; Beckman Coulter, USA) and topped up with 0.1 µm‐filtered PBS (Corning) if necessary, and processed using an Optima XE‐90 ultracentrifuge equipped with an SW55‐Ti swinging‐bucket rotor (Beckman Coulter).

### 2.6 Nanoparticle Tracking Analysis

The particle content of samples (diluted in PBS) was measured using a NanoSight NS500 instrument (488 nm laser) with NTA software, version 3.4 (Malvern Panalytical, UK). For every sample, five 60‐s videos were recorded at an infusion rate at 15 and camera brightness level at 12 or 13. The mean particle concentration and size per sample were calculated.

### 2.7 Western Blotting

Following ultracentrifugation, sEVs were lysed with RIPA lysis buffer containing protease inhibitors (Roche, Switzerland), and protein concentration was determined using a Pierce BCA protein assay kit (Thermo Fisher Scientific). For each sample, 7 µg of protein was separated on a NuPAGE 4%–12% Bis‐Tris Gel (Thermo Fisher Scientific) and transferred to a methanol‐activated PVDF membrane. Transfer and protein loading were verified with Ponceau S (Sigma–Aldrich) staining. Membranes were blocked with 5% Blotto (Alpha Diagnostic International, USA) in PBS with 0.05% Tween‐20 for 1 h at room temperature and incubated overnight at 4°C with shaking with antibodies directed towards Syntenin (Abcam) and albumin (Cell Signalling, USA) or argonaute‐1 (Cell Signalling). After washing three times with PBS with 0.05% Tween‐20, the membrane was incubated for 1 h with swine anti‐rabbit‐IgG HRP‐conjugated secondary antibodies (Dako, USA) at room temperature. Protein bands were visualised with EZ‐ECL (Biological Industries) and imaged using a Gel‐Box system (Syngene, India). Details of all antibodies used are listed in Table S2.

### 2.8 Transmission Electron Microscopy

For EV visualisation, a carbon‐coated 300 mesh copper grid was glow discharged and then incubated on a 10 µL droplet of the sample for 2 min, blotted with filter paper, negatively stained with 2% uranyl acetate for 10 s, blotted and air dried. Grids were imaged at an accelerating voltage of 120 kV in an FEI T12 transmission electron microscopy (TEM) using a Gatan OneView digital camera.

### 2.9 Multiplex Bead‐Based Flow Cytometry

sEV surface proteins were characterised using the MACSPlex EV kit IO (Miltenyi Biotec, Germany) following the manufacturer's instructions. The kit is a microbead‐based technique, allowing the detection of 37 sEV surface epitopes. Samples of PF‐sEV were analysed at a concentration of 6.25 × 10^9^ EVs/mL (within the range of physiological concentrations), and EEO‐sEVs were analysed at a concentration of 1 × 10^9^ EVs/mL, while pMΦ‐sEVs were assessed at 2 × 10^8^ EVs/mL (concentrations were determined by titration within values chosen in the linear range and beneath bead saturation points, data not shown) and using NTA). Briefly, 120 µL of sEV suspension in PBS was incubated overnight with 15 µL of capture beads. Following incubation, a mixture of APC‐conjugated detection antibodies specific to tetraspanins (–CD9, CD63 and CD81) was added.

As a negative control, capture beads were incubated with detection antibodies in PBS alone. Distinct bead populations binding different surface epitopes were discriminated by their respective fluorescence intensities during flow cytometry analysis. The median fluorescence intensity (MFI) data were normalised following the manufacturer's guidelines. Background fluorescence was accounted for by subtracting the MFI of the buffer‐only control. Any marker with a signal below that of the corresponding isotype control was excluded from further analysis. Sample MFIs were subsequently normalised by dividing the mean MFI of CD9, CD63 and CD81 in each sample by the overall mean MFI of these markers across all samples.

CD47 is not a marker included in the original MACSPlex EV kit IO. To detect the coexpression of CD47 with other markers in the MACSPlex EV panel on PF‐sEVs from control (*n* = 5) and EM (*n* = 5) patients, a modified MACSPlex EV Kit IO protocol (Görgens et al. 2021) was applied. In this modified protocol, 5 µL of antibody towards human CD47‐APC conjugation (clone CC2C6, BioLegend, USA) was used in place of the standard incubation with detection antibodies. Coexpression was assessed by calculating fold change values, defined as the ratio of MFI from EV‐positive beads to that from PBS‐only beads. A marker was considered positively coexpressed with CD47 if ≥3 out of five samples in a group showed a fold change greater than 1. Flow cytometry acquisition was performed using a BD LSR II (BD Biosciences, USA) for all PF‐derived experiments or a Cytek Northern Lights spectral flow cytometer (Cytek Biosciences, USA) for all EEO‐derived experiments.

### 2.10 THP‐1 Cell Culture and Differentiation

THP‐1 cells were purchased from ATCC and STR profiled. THP‐1 cells were cultured in RPMI‐1640 media (Sigma–Aldrich) with 10% FCS, 0.05 mM 2‐mercaptoethanol, 100 U/mL penicillin and 100 µg/mL streptomycin. Cells were grown at 37°C with 5% CO_2_ at a concentration of 4 × 10^5^ to 2 × 10^6^ up to 30 passages. For experiments, a density of 1 × 10^6^ cells/mL was used. THP‐1 cells were differentiated to THP‐1 derived macrophages with 5 ng/mL phorbol 12‐myristate 13‐acetate (PMA) (Sigma–Aldrich) for 48 h and were rested in PMA‐free complete growth media for 24 h. THP‐1 derived macrophages were treated with 20 ng/mL interleukin 4 (IL‐4) (Peprotech, USA) and 20 ng/mL interleukin 13 (IL‐13) (Peprotech) for 48 h to differentiate into M2 macrophages. Cells were stained with anti‐CD206 (Mouse IgG1, κ, clone 15‐2, APC, BioLegend) and anti‐CD163 (Mouse IgG1, κ, clone GHI/61, Brilliant Violet 711, BioLegend) antibodies, then analysed using flow cytometry (BD LSR II, BD Biosciences, USA). Mean fluorescence intensity (MFI) was quantified and expressed as fold change relative to undifferentiated THP‐1 cells.

### 2.11 Confocal Microscopy Analysis of sEVs Uptake and Phagocytic Activity in Macrophages

Pooled sEV‐enriched fractions isolated via qEV SEC (IZON, New Zealand) were stained using the PKH26 Red Fluorescent Cell Linker Kit (Sigma–Aldrich). Briefly, 2 µL of PKH26 dye was incubated with the sEV fraction in 1 mL Diluent C for 5 min at room temperature. The reaction was stopped with 1 mL of 1% BSA (Sigma–Aldrich) in PBS, followed by ultracentrifugation at 150,000 × *g* for 2 h at 4°C. A control sample without sEVs underwent the same staining and ultracentrifugation to assess nonspecific fluorescence.

Both sEVs and control pellets were resuspended in 20 µL fPBS and incubated overnight with THP‐1 macrophages (2 × 10^5^ cells/well) on 8‐well chamber slides (ibidi, Thermo Fisher Scientific). Macrophages were then treated with 20 µg/mL *Escherichia coli* Deep Red pHrodo bioparticles (Thermo Fisher Scientific) for 1 h, washed with PBS and stained with wheat germ agglutinin (WGA) conjugated to Alexa Fluor 488 (Thermo Fisher Scientific) for 10 min at 37°C. After fixation with 4% formaldehyde, permeabilisation with 0.5% Tween‐20 (Sigma–Aldrich) and mounted in mounting media with DAPI (Abcam), phagocytosis was visualised using an LSM 900 Airyscan 2 confocal microscope (Zeiss, Germany).

### 2.12 Flow Cytometry Analysis of Phagocytic Activity in Macrophages

Macrophage phagocytic activity was titrated using 0–50 µg/mL *E. coli* Deep Red pHrodo bioparticles (Thermo Fisher Scientific) and measured by flow cytometry after 1 h incubation to determine the optimal concentration for subsequent experiments.

To assess the effect of sEVs on phagocytic activity, macrophages (THP‐1 derived M2 macrophages and pMΦ, 2 × 10^5^ cells/well in 48‐well plates) were pretreated with either 10^10^ EVs/mL PF‐sEVs or 10^9^ EVs/mL EEO‐sEVs (concentration confirmed by NTA) for 24 h before 20 µg/mL bioparticle exposure. For EV‐depleted controls, THP‐1‐derived macrophages were treated with 5% PF or EV‐depleted PF (following ultracentrifugation at 150,000 × *g* for 4 h at 4°C) or with EEO CM after 72 h incubation or EV‐depleted CM. Controls included macrophages treated with bioparticles alone (negative gating control) and macrophages pretreated with 10 µM cytochalasin D (Sigma–Aldrich) for 10 min before bioparticle addition (inhibition control). Following bioparticle incubation, macrophages were washed with PBS and detached using Accutase (Sigma–Aldrich) for 10–30 min at 37°C. Cells were then immediately placed on ice to halt phagocytosis, washed with 2% FCS in PBS (300 × *g*, 5 min, 4°C) and resuspended in PBS. CD47 blocking antibody (5 µg, clone B6H12, Thermo Fisher Scientific) or mouse IgG control (MOPC‐21, Biolegend) were incubated with sEVs overnight at 4°C then were washed with PBS using ultracentrifugation before preincubation with macrophage for 24 h before performing the pHrodo bioparticle experiment as described.

Phagocytic activity was quantified by flow cytometry using a BD LSR II (BD Biosciences, USA) for all PF‐derived experiments or a Cytek Northern Lights spectral flow cytometer (Cytek Biosciences) for all EEO‐derived experiments. The frequency of the Deep Red PHrodo positive cells and/or the MFI were determined. For THP‐1 macrophages, experimental MFIs were expressed as fold change relative to the mean MFI from triplicate wells of bioparticle‐only controls.

### 2.13 Statistical Analysis

For multiple group comparisons, one‐ or two‐way ANOVA with Tukey's post‐hoc test was applied. Two‐tailed Student's *t* tests were used to compare two group means. Normality was assessed using the Shapiro–Wilk test, with nonparametric alternatives used for nonnormal data. Throughout all analyses, *p* < 0.05 was considered statistically significant. Spearman's rho correlation coefficient (*r*) was used to assess correlations among surface markers expression on PF‐sEVs.

EEO‐sEVs and PF‐sEVs MACSPLEX data were analysed by unsupervised clustering using R. Selected markers for hierarchy clustering were normalised using row‐wise *z* score transformation. For EEO‐sEVs, Euclidean distance with Ward.D2 linkage was used for sample (column) clustering and Spearman correlation distance with Ward.D2 linkage for marker (row) clustering. For PF‐sEVs, both row and column clustering used Spearman correlation distance and Ward.D2 linkage to account for greater sample heterogeneity. Clustering robustness was assessed by cophenetic correlation coefficient and between‐group molecular divergence was calculated using Euclidean distances. Visualisation and analysis were conducted using the ‘pheatmap’ R package. Principal component analysis (PCA) was performed using the prcomp function in R, with samples as observations and MACSPLEX EV marker as variables. The data matrix was standardised by centring and scaling to unit variance. Group‐wise clustering was visualised using 95% confidence ellipses. The Top 10–15 contributing features for each principal component were identified based on their absolute loading values. Group differences among PC1 and PC2 were assessed using Kruskal–Wallis tests followed by pairwise Wilcoxon rank‐sum tests with Benjamini–Hochberg correction for multiple comparisons.

## 3 Results

### 3.1 Endometrial Epithelial Organoids Serve as a Reliable Model for Extracellular Vesicle Studies in Endometriosis

**FIGURE 1:**
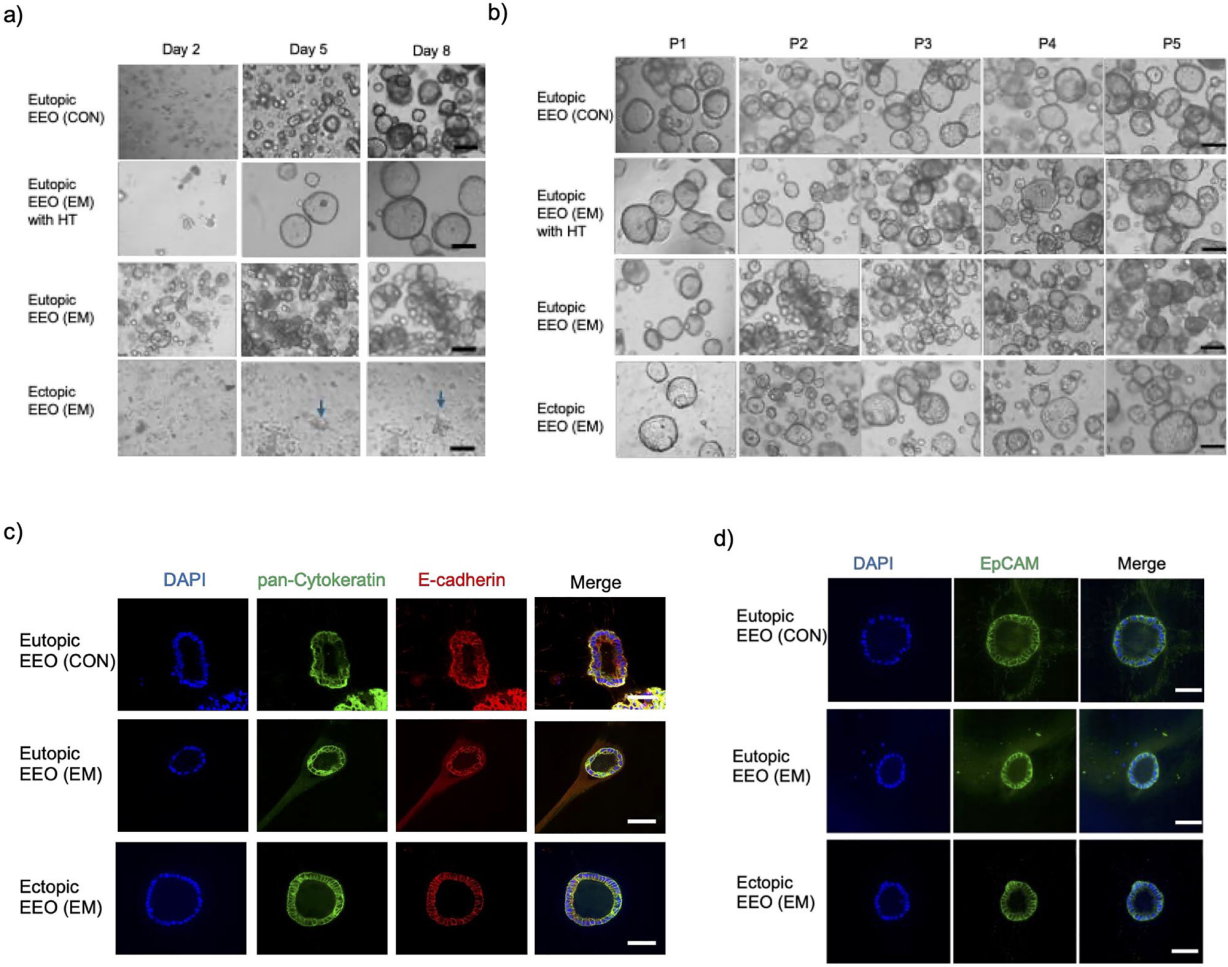
Growth comparison of EEO from Passages 0 to 5. (a, b) Representative bright‐field microscopy images showing the development of EUT EEO from EM and CON and also women with HT and ECT EEO from EM (a) at Days 2, 5 and 8 postestablishment at P0 and (b) at Day 5 at P1–P5. Scale bar, 100 µm. Blue arrows indicate organoid‐like structure in ECT EEO. (c, d) Representative confocal microscopy images of EUT EEO from EM and CON patients, and EUT EEO from EM at P3. Samples were stained for (c) epithelial markers pan‐cytokeratin (green) and E‐cadherin (red) and (d) endometrial marker EpCAM (green). Nuclei were stained with DAPI (blue). Scale bar, 50 µm. CON, control; ECT, ectopic epithelium; EEO, endometrial epithelial organoid; EM, endometriosis; EUT, eutopic epithelium; HT, hormonal treatment.

We established three types of endometrial epithelial organoids (EEOs). ECT EEOs were derived from peritoneal lesions of EM patients. EUT EEOs were established from two sources: the endometrium of women diagnosed with EM [with and without hormone treatment (HT)] and the endometrium of women without EM (controls, CON). Patient details are shown in Table 1. At Passage 0 (P0), tissue from ECT peritoneal lesions required longer dissociation times and exhibited slower formation of EEO. No differences in EEO formation were observed between EUT samples from EM and CON. However, fewer EEOs were obtained from EUT samples collected from women undergoing HT (Figure 1a). No differences in morphology or growth were observed after passaging (P1–P5) in ECT EEO or EUT EEO from different patients (EM, CON or women on HT) (Figure 1b). This observation is consistent with findings reported by Boretto et al. (2019). All three EEO types were positive for pan‐cytokeratin and E‐cadherin, confirming their epithelial origins (Figure 1c). EpCAM expression further confirmed the endometrial lesion origin of ECT EEO (Figure 1d), as background mesothelial cells in peritoneum are EpCAM‐negative (Yonemura et al. 2024).

**TABLE 1:** Patient characteristics and sample information for eutopic endometrium and endometriosis lesion samples.

Patient ID	EM staging (0–4)	EEO type			Menstrual cycle	Hormone use	Age (years)	BMI
		EUT	ECT	ECT location				
1	0	Y	N	N/A	Secretory	N	39	N/A
2	0	Y	N	N/A	Proliferative	N	21	39
3	0	Y	N	N/A	Proliferative	N	23	20
4	0	Y	N	N/A	Proliferative	N	23	27
5	2	Y	Y	Ovarian fossa	Secretory	Y	34	32
6	2	Y	N	N/A	Secretory	N	40	25
7	3	Y	N	N/A	Proliferative	N	26	24
8	2	Y	Y	Ovarian fossa	N/A	N	23	27
9	4	Y	Y	Peritoneal sidewall	Proliferative	N	24	32
10	1	Y	Y	Pouch of Douglas	N/A	Y	18	19
11	2	Y	Y	Peritoneal sidewall	Secretory	N	26	37
12	2	N	Y	Ovarian fossa	N/A	Y	47	26
13	4	N	Y	Recto‐vaginal	N/A	Y	38	43

*Note*: EM staging (0–4) is classified according to rASRM guidelines (Mallegol et al. 2007).

Abbreviations: ECT, ectopic; EM, endometriosis; EUT, eutopic; N, no; N/A, not applicable/available; Y, yes.

**FIGURE 2:**
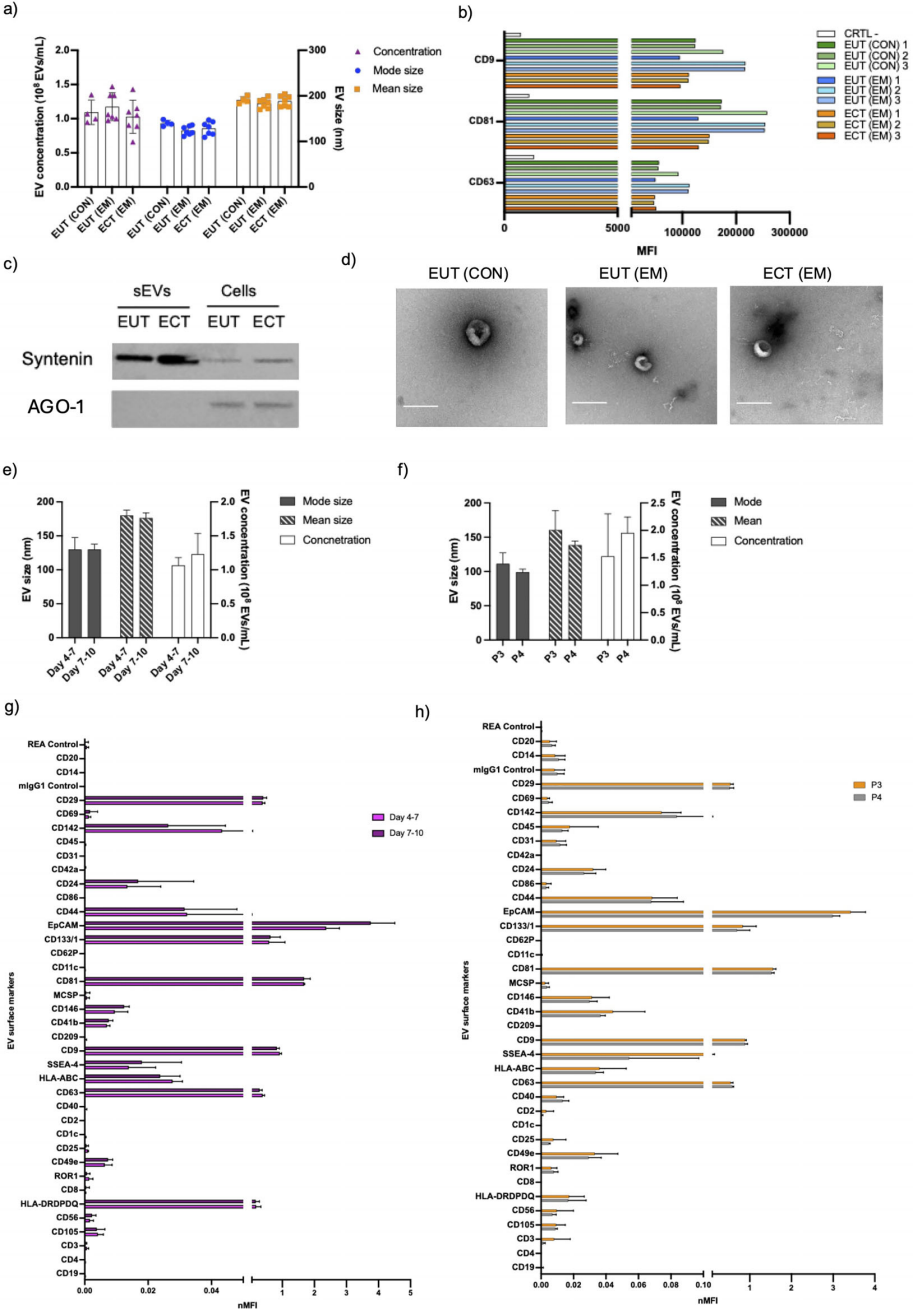
Characterisation of EEO‐sEVs. (a) The mode size, mean size and concentration of EEO‐sEVs derived from the EUT of CON and EM patients, and the ECTs of EM were analysed by NTA. Data are shown as mean ± SD. (b) Flow cytometry analysis of CD81, CD9 and CD63 expression in EEO‐sEVs derived from EUT (CON), EUT (EM) and ECT (EM) (*n* = 3). Marker expression was quantified by MFI. (c) Western blot analysis of syntenin and AGO‐1 (argonaute‐1) expression in pooled EUT (CON + EM) and ECT (EM) EEO‐sEVs (*n* = 3) compared to EEO cell lysates. (d) Representative TEM image of EEO‐sEVs derived from EUT (CON) and EUT (EM) and ECT (EM). Scale bar, 200 nm. (e, f) NTA of mode size, mean size and concentration of EVs populations isolated from EEO (EUT EM and ECT EM) collected from 72 h incubation (e) at Days 4–7 and 7–10 at P3 (*n* = 3) and (f) at P3 and P4 (*n* = 4). Data are shown as mean ± SD. (g, h) Surface marker profiles of EEO‐sEVs (EUT EM and ECT EM) collected from 72 h incubation at (g) Days 4–7 and 7–10 at Passage 3 (P3) (*n* = 3), and (h) at P4 was analysed by flow cytometry. The MFI (MACSPlex) was normalised by average MFI of CD81, CD9 and CD63. Data are shown as mean nMFI ± SD. ARG‐1, argonaute‐1; CON, control; ECT, ectopic epithelium; EEO, endometrial epithelial organoid; EM, endometriosis; EUT, eutopic epithelium; MFI, median fluorescence intensity; sEV, small extracellular vesicle.

sEV isolation was performed by ultracentrifugation and SEC at P3 and P4. sEVs were released from all three EEO types, no significant differences in secreted EV mode size (123 ± 9, 140 ± 6 and 128 ± 15 nm; mean ± SD) or concentration (1.18 × 10^8^ ± 2.04 × 10^7^, 1.09 × 10^8^ ± 1.78 × 10^7^, 1.03 × 10^8^ ± 2.43 × 10^7^ EVs/mL; mean ± SD) were observed among EUT EEO from EM patients, controls and ECT EEO, respectively (Figure 2a). We also examined sEV characteristics in relation to HT status and found no obvious differences (data not shown), therefore, these samples were analysed collectively in downstream analyses (Table 1).

EVs were further characterised by the identification of the recognised sEV‐enriched markers: CD9, CD81 and CD63 by bead‐based flow cytometry analysis (Figure 2b), expression was similar within and between groups. Syntenin protein enrichment in sEV compared to cells from EUT‐ and ECT‐EEO was confirmed by immunoblotting (Figure 2c). Purity was confirmed by the reduction of nonvesicular protein Argonaute‐1 (Figure 2c). sEV morphology and a clear background indicating minimal soluble protein contamination in preparations were confirmed by TEM (Figure 2d).

sEV preparations showed consistency in size, concentration across collection times (Days 4–7 or 7–10) in P3 (Figure 2e) and between passage number (P3 and P4) (Figure 2f), demonstrating the EEO model's temporal stability for EV production. The expression of 37 surface markers on EVs remained stable across collection times Days 4–7 versus 7–10 of culture (Figure 2g) and Passages 3 versus 4 (Figure 2h). sEV preparations collected from P2 showed variability in the expression of seven different markers (Figure S1), possibly due to residual background cells, therefore only sEVs from P3 to P5 were used in subsequent experiments.

### 3.2 Ectopic Endometrial Epithelial sEVs Exhibit a Lesion Specific Surface Marker Profile

Having confirmed the consistent production of sEVs across passages and collection times in EEO cultures, we next performed phenotyping of sEVs derived from ECT and EUT EEO by assessing the expression of 37 surface proteins using semi‐quantitative multiplex bead‐based flow cytometry (MACSPlex EV kit IO). The expression level of each marker was measured as the normalised median fluorescence intensity (nMFI) of CD9+, CD63+ and CD81+ sEVs. A total of 22 protein markers were present across all EEO‐sEV samples (Table 2). Fifteen proteins were excluded from further analysis due to low detection, with measured nMFIs near or below the detection threshold (Table S3). CD133/1 and EpCAM were the most abundant markers, higher than the expression of sEV enriched tetraspanins, CD81, CD9 and CD63, indicating their potential utility as cell specific markers of EE cell originated sEVs. Additionally, proteins associated with immunomodulation (HLA‐DRDPDQ, CD29, CD24) and stem cell properties (ROR‐1, SSEA‐4) were detected.

**TABLE 2:** Surface marker expression on EEO‐sEVs measured by multiplex bead‐based flow cytometry.

	EUT (CON)		EUT (EM)		ECT (EM)		Group difference	
	Mean	SD	Mean	SD	Mean	SD	*p* value	*F* value
CD3	0.0017	0.0008	0.0015	0.0005	0.0013	0.0009	0.77	0.30
CD105	0.0046	0.0008	0.0056	0.0015	0.0068	0.0018	0.18	2.00
CD56	0.0022	0.0008	0.0022	0.0004	0.0029	0.0017	0.46	0.80
HLA‐DRDPDQ	0.0519	0.0665	0.0622	0.0776	0.0888	0.0956	0.73	0.30
ROR1	0.0037	0.0016	0.0047	0.0041	0.0101	0.0110	0.38	1.00
CD49e	0.0082	0.0017	0.0099	0.0036	0.0112	0.0063	0.82	0.20
CD25	0.0013	0.0006	0.0013	0.0004	0.0012	0.0005	0.67	0.40
CD40	0.0056	0.0023	0.0087	0.0111	0.0226	0.0300	0.19	1.90
CD63	0.4845	0.0260	0.5364	0.0447	0.5602	0.0542	0.08	3.10
HLA‐ABC	0.0102	0.0064	0.0186	0.0091	0.0220	0.0124	0.25	1.50
SSEA‐4	0.0687	0.0332	0.0967	0.0600	0.0857	0.0672	0.83	0.20
CD9	0.9780	0.0482	0.9153	0.0993	0.9551	0.1237	0.63	0.48
CD41b^*^	0.0094	0.0035	0.0117	0.0046	0.0049	0.0025	0.01^*^	6.10
CD146	0.0405	0.0211	0.0609	0.0237	0.0804	0.0382	0.13	2.3
CD81	1.5375	0.0630	1.5485	0.0715	1.4847	0.0998	0.45	0.90
CD133/1	3.7786	0.6223	2.7880	0.6956	4.1997	1.6926	0.13	2.40
EpCAM^*^	3.1367	0.2012	2.3469	0.4899	2.3339	0.3726	0.01^*^	6.18
CD44^*^	0.0723	0.0322	0.0912	0.0152	0.1277	0.0337	0.01^*^	5.95
CD24	0.1442	0.0391	0.1094	0.0604	0.1057	0.0481	0.48	0.70
CD142	0.0414	0.0251	0.0680	0.0229	0.0751	0.0269	0.22	1.80
CD69	0.0096	0.0074	0.0098	0.0160	0.0097	0.0038	0.99	0.00
CD29^*^	0.4887	0.0999	0.4800	0.0556	0.6397	0.1174	0.01^*^	6.00

*Note*: Normalised median fluorescent intensity (nMFI) values (mean and SD) are presented for each group: EUT EEO from CON (*n* = 4), EM (*n* = 7) and ECT EEO from EM (*n* = 7). *p* values and *F* values from one‐way ANOVA are provided for each marker.

Abbreviations: CON, control; ECT, ectopic; EM, endometriosis; EpCAM, epithelial cell adhesion molecule; EUT, eutopic; HLA‐ABC, human leukocyte antigen‐ABC; HLA‐DRDPDQ, human leukocyte antigen‐DRDPDQ; ROR1, receptor tyrosine kinase‐like orphan receptor 1; SSEA‐4, stage‐specific embryonic antigen‐4.

* Significant differences between groups (*p* < 0.05) are indicated.

**FIGURE 3:**
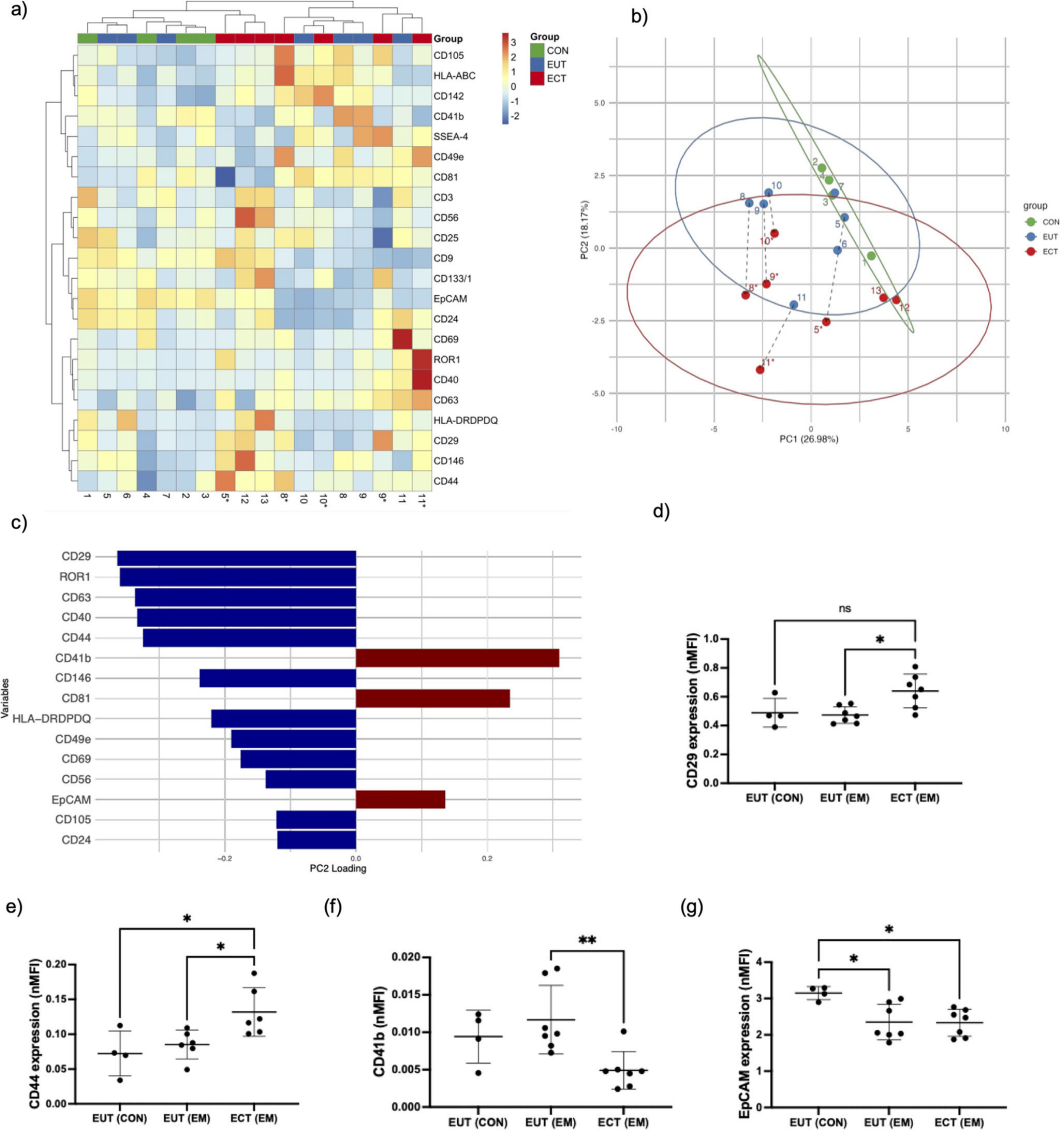
ECT EEO has distinct EV surface profiles (MACSPlex analysis). (a) Heatmap indicating hierarchical clustering analysis of nMFI surface marker expression on EEO‐sEV among EUT (CON), EUT (EM) and ECT (EM). The MFI was normalised by average MFI of CD81, CD9 and CD63. Patient IDs are indicated at the bottom; * denotes paired ECT EEO‐sEVs samples from the same EM patient. (b) PCA of surface marker profiles in EEO‐sEVs. PC1 versus PC2 scores plot showing sample distribution across CON (green), EUT (blue) and ECT (red) groups. Ellipses represent 95% confidence intervals. Paired EUT and ECT samples are indicated by matching numbers with * on ECT, connected by black dashed arrows. (c) Top 15 surface markers contributing to PC2 variance in the PCA. Positive loadings (red) indicate markers with higher expression in the positive direction of PC2, while negative loadings (blue) indicate markers with higher expression in the negative direction. The length of each bar represents the magnitude of contribution. (d–g) The nMFI of (d) CD29, (e) CD44, (f) CD41b and (g) EpCAM expression on EEO‐sEVs from EUT (CON), EUT (EM) and ECT (EM). Data are presented as mean ± SD. *p < 0.05, **p < 0.01. CON, control; ECT, ectopic epithelium; EEO, endometrial epithelial organoid; EM, endometriosis; EUT, eutopic epithelium; MFI, median fluorescence intensity; sEV, small extracellular vesicle.

Hierarchical clustering showed partial separation between ECT and CON, while EUT EEO samples appeared to bridge the two groups (Figure 3a). Paired EUT and ECT EEO samples clustered together in some cases but not all. Similarly, PCA revealed partial clustering by EEO type, with PC1 and PC2 accounting for 26.98% and 18.17% of the variance, respectively (Figure 3b). Significant separation along PC2 was observed (Kruskal–Wallis test, *p* = 0.012), with ECT EEO (−1.80 ± 1.41) differing from both CON EEO (1.66 ± 1.34) and EUT EEO (0.85 ± 1.41). EUT EEO samples again appeared intermediate between CON and ECT profiles. Within paired samples, ECT EEO consistently showed a downward shift along PC2 relative to EUT EEO, suggesting coordinated changes. The strongest negative contributors to PC2 included CD29, CD44, ROR1, CD40 and CD146, while EpCAM, CD41b and CD81 were the positive contributors (Figure 3c).

Differential expression analysis confirmed the PCA observations, revealing significant variations in CD44, CD29, CD41b and EpCAM levels between the three EEO types (*p* < 0.05) (Table 2). Post‐hoc analysis revealed CD29 expression was significantly higher in sEVs from ECT EEO (EM) compared to EUT EEO (EM) (*p* < 0.05) (Figure 3d). Similarly, CD44 expression was significantly elevated in ECT (EM) compared to both EUT (EM) and EUT EEO (CON) (*p* < 0.05) (Figure 3e). In contrast, CD41b expression was significantly lower in ECT EEO than those in EUT EEO (EM) (*p* < 0.01) (Figure 3f). Additionally, EpCAM expression was significantly reduced in sEVs from both ECT EEO (EM) and EUT EEO (EM) compared with EUT EEO (CON) (*p* < 0.05) (Figure 3g). These markers are likely reflecting adaptation in the peritoneal microenvironment, whereas altered expression of adhesion molecules (CD29, CD44), angiogenesis (CD146/CD142), immune modulators (CD41b, CD40) and cellular phenotype markers (EpCAM) may facilitate lesion establishment and survival.

### 3.3 Endometrial Epithelial‐sEVs Are Abundant in the Peritoneal Microenvironment

Following *in vitro* characterisation of EE sEVs derived from ECT‐EEO, we investigated their presence in PF. PF has previously been shown to exhibit a distinct sEV protein profile in EM patients (Nazri et al. 2020). Building on this knowledge, we examined cell origins of PF‐sEVs and investigated lesion‐EE‐specific signatures to validate our in vitro findings in the PF environment.

**FIGURE 4:**
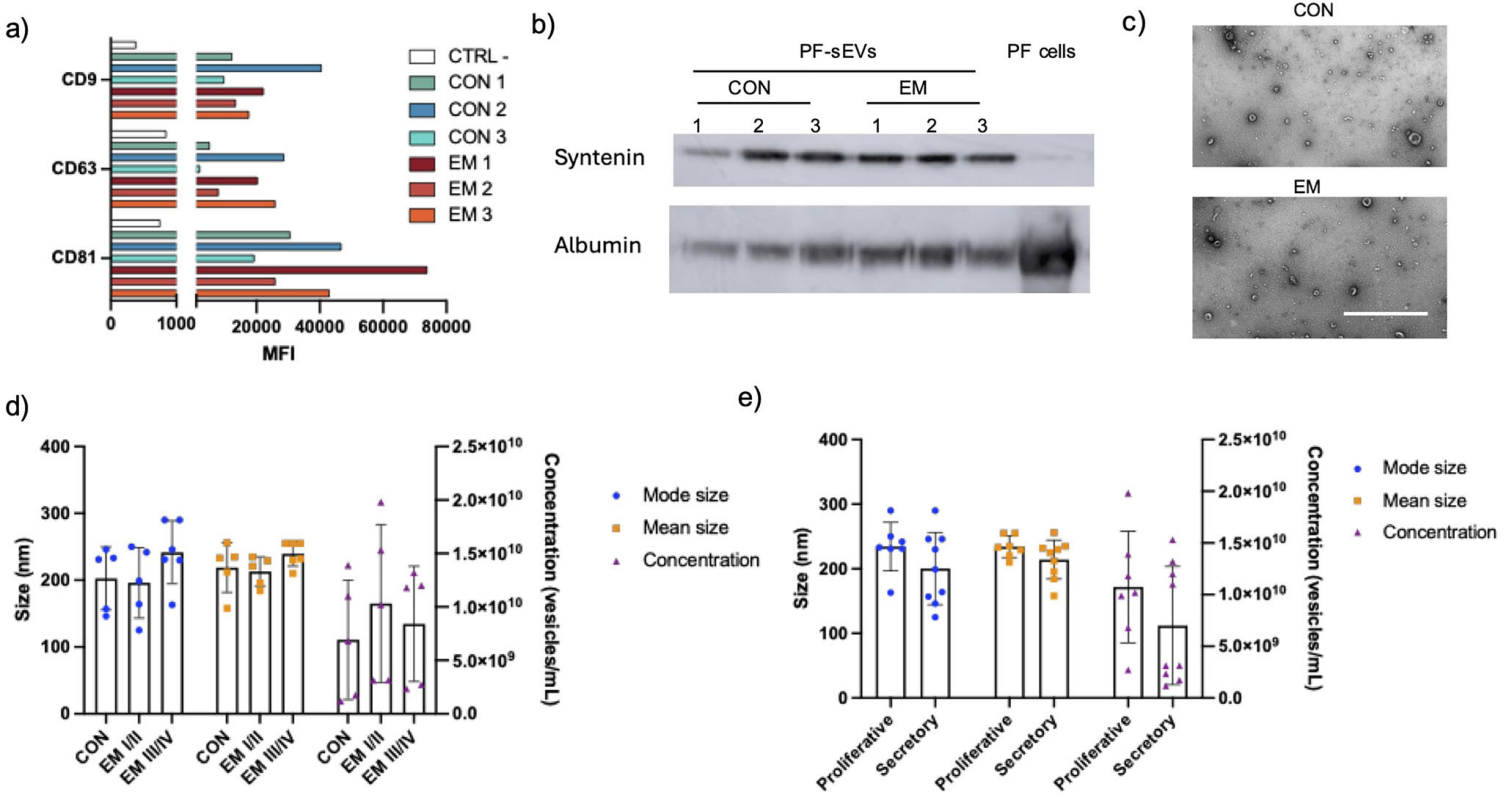
PF‐sEVs are derived from various cell origins. (a) MFI of CD81, CD9 and CD63 expression on sEVs isolated from the PF of individual CON and EM patients. CTRL represents the negative control of MACSPLEX capture beads incubated with PBS with no PF‐sEVs. (b) Western blot analysis of syntenin and albumin expression in EEO‐sEVs isolated from CON and EM (*n* = 3) compared to PF cell lysates. (c) Representative TEM image of PF‐sEVs derived from CON and EM. Scale bar, 1000 nm. (d, e) NTA of PF‐sEVs shows the mode size, mean size and concentration of PF‐sEVs derived from CON, EMI/II and EMIII/IV stage patients (d) and proliferative versus secretory phase of the menstrual cycle (e). CON, control; EM, endometriosis; MFI, median fluorescence intensity; PF, peritoneal fluid; pMΦ, peritoneal macrophage; sEV, small extracellular vesicle.

PF‐sEVs were assessed by the expression of sEV‐enriched markers: CD9, CD81 and CD63 by bead‐based flow cytometry analysis (Figure 4a), expression was similar within and between control and EM groups. Syntenin protein was enriched in sEV compared to PF resident cells as demonstrated by immunoblotting (Figure 4b). Purity was confirmed by reduced albumin expression (Figure 4b) combined with TEM image verification of EV morphology with minimal protein background (Figure 4c).

PF‐sEVs were isolated from PF collected from women (*n* = 16) with EM and without (CON), none of whom had received hormonal treatment in the 3 months before surgery. NTA revealed a concentration range from 1.2 × 10^9^ to 2.0 × 10^10^ EVs/mL, with an average of 8.6 × 10^9^ ± 5.7 × 10^9^ EVs/mL (mean ± SD, *n* = 16) (Table 3). Statistical analysis revealed no significant influence of either disease status (rASRM staging) or menstrual cycle phase (proliferative versus secretory) on the concentration and size of PF‐sEVs (Figure 4d, e). The observed variability in particle size (215 ± 50 nm, mean ± SD, *n* = 16) likely reflects the heterogeneous origins and dynamic change of sEVs within the peritoneal microenvironment. This observation is consistent with a previous report using different isolation methods that demonstrated broad size distributions rather than a sharp, single peak profile on NTA (Nazri et al. 2020).

**TABLE 3:** Baseline patient information and corresponding PF‐sEV concentration and mode size measured by NTA.

Patient ID	EM staging (0–4)	Menstrual cycle	Age	BMI	Concentration (EVs/mL)	Mode size (nm)
1	0	Secretory	32	23	1.17E+09	146
2	0	Secretory	38	21	1.75E+09	246
3	0	Secretory	23	19	1.10E+10	157
4	1	Secretory	40	25	1.53E+10	164
5	2	Secretory	40	24	3.14E+09	125
6	4	Secretory	33	25	2.34E+09	246
7	0	Proliferative	38	33	1.39E+10	233
8	0	Proliferative	27	18	6.80E+09	231
9	4	Proliferative	31	22	1.18E+10	231
10	2	Proliferative	28	22	1.98E+10	250
11	2	Proliferative	30	22	1.02E+10	242
12	3	Proliferative	29	24	9.90E+09	290
13	4	Proliferative	43	22	2.72E+09	163
14	2	Secretory	25	24	3.13E+09	199
15	3	Secretory	40	24	1.20E+10	230
16	4	Secretory	29	20	1.32E+10	290

Surface marker profiling of PF‐sEVs using multiplexed bead‐based flow cytometry was performed on 35 markers, after excluding CD209 and CD1c due to low prevalence (positive in <20% samples) and nMFI values (<0.01). Among the analysed markers, the highest nMFI values (mean ± SD) were observed for CD81 (1.69 ± 0.19), CD24 (0.82 ± 0.52), CD9 (0.80 ± 0.14), CD133/1 (0.55 ± 0.72), CD63 (0.49 ± 0.19) and EpCAM (0.41 ± 0.44) (Table S5).

**FIGURE 5:**
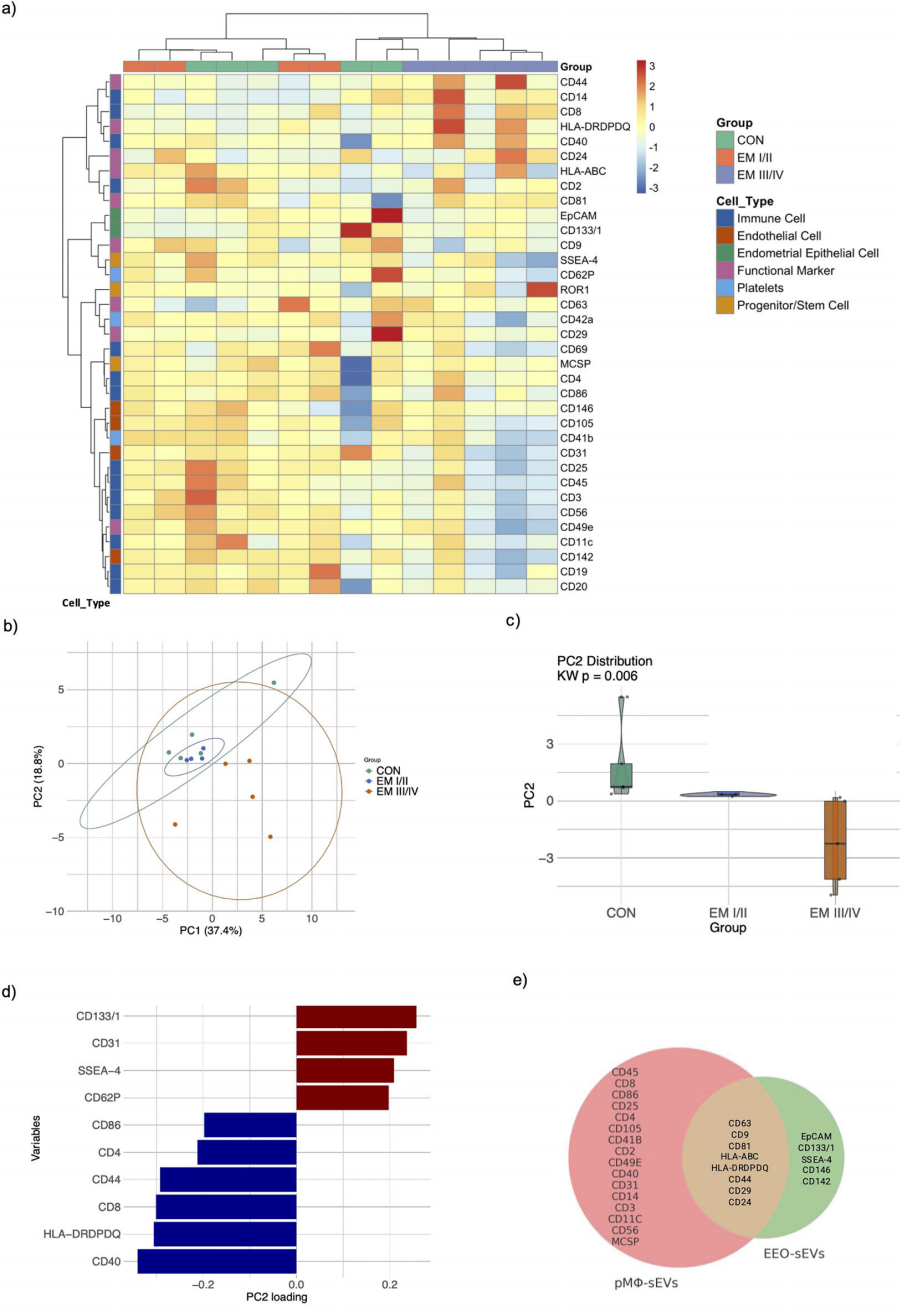
PF‐sEV surface marker characterisation (MACSPlex analysis). (a) Heatmap indicating hierarchical clustering analysis of nMFI surface marker expression on PF‐sEVs. (b) PCA of surface marker profiles in PF‐sEVs. PC1 versus PC2 scores plot showing sample distribution across CON (blue), EM I/II (green) and EM III/IV (red) groups. Ellipses represent 95% confidence intervals. (c) Violin plots showing the distribution of PC2 scores across groups. Black bars indicate median values and quartiles. (d) Top 10 surface markers contributing to PC2 variance in the PCA. Positive loadings (red) indicate markers with higher expression in the positive direction of PC2, while negative loadings (blue) indicate markers with higher expression in the negative direction. The length of each bar represents the magnitude of contribution. (e) Venn diagram showing the distribution and overlap of surface markers on sEVs derived from pMΦ and EEO. CON, control; EEO, endometrial epithelial organoid; EM, endometriosis; nMFI, normalised median fluorescence intensity; PCA, principal component analysis; PF, peritoneal fluid; pMΦ, peritoneal macrophage; sEV, small extracellular vesicle.

Marker clustering analysis suggested multiple cellular sources of PF‐sEVs (Figure 5a). The analysis revealed distinct clusters of EE markers (EpCAM/CD133/1), endothelial markers (CD105/CD146, CD31/CD142) and B cell markers (CD20/CD19). Several clusters of T cell markers, such as CD3/CD25/CD45/CD56, CD4/CD86 and CD40/CD8/CD14, appeared to correlate with antigen‐presenting cell populations. The presence of stem cell markers SSEA‐4/ROR‐1 and functional markers associated with migration, adhesion and immunomodulation (HLA‐DRDPDQ, HLA‐ABC, CD44, CD24 and CD29) was also observed.

Sample clustering through hierarchical analysis demonstrated that EM III/IV clustered separately and showed divergent PF‐sEV profiles compared to CON and EM I/II, with CON PF‐sEV profiles showing the largest variance. PCA analysis provided additional support for these findings (Figure 5b).

PCA revealed two major components explaining 56.23% of the total variance (PC1: 37.44% and PC2: 18.79%). Although PC1 showed no significant differences between groups (Figure S5), PC2 demonstrated significant lower scores in EM III/IV samples compared to EM I/II and CON groups (Kruskal–Wallis test, *p* = 0.006) (Figure 5c). PC2 was characterised by negative correlations with immune markers (CD40, CD8, HLA‐DRDPDQ, CD4, CD44 and CD86) and positive correlations with stem cell and endothelial markers (CD133/1, SSEA‐4, CD62P and CD31) (Figure 5d). This pattern suggests that EM III/IV is associated with enhanced immune marker expression and reduced stem cell marker expression on sEV.

Given that PF‐sEVs also have high expression of immune markers and pMΦs represent the predominant immune cell type in PF, we next assessed sEVs derived from pMΦ as a comparison. pMΦs were isolated according to a previously described protocol (Lee et al. 2021) and confirmed by morphology and surface markers (CD14^+^/CD45^+^/EpCAM^−^/CD10^−^/CD3^−^) (Figure S2). sEVs from pMΦ were characterised by NTA and TEM (Figure S3). Bead‐based flow cytometry revealed a distinct immune‐enriched profile, with 27 markers (including CD9, CD63 and CD81) expressed across all samples (*n* = 19), among which HLA‐DRDPDQ was the most abundantly expressed marker on pMΦ‐sEVs (Table S4).

The positive marker expression from in vitro culture of EEO‐sEVs and pMΦ‐sEVs across all samples was visualised using a Venn diagram (Figure 5e). This analysis revealed enrichment of markers consistent with the parental cell types, with CD45 and CD14 solely expressed in pMΦ‐sEVs, whereas EpCAM and CD133/1 were exclusively detected in EEO‐sEVs. Apart from sEV‐enriched markers (CD9, CD63 and CD81), five functional markers are shared among EEO‐sEVs and pMΦ‐sEVs, including HLA‐DRDPDQ, HLA‐ABC, CD24, CD44 and CD29.

Although multivariate analysis revealed distinct patterns of PF‐sEV marker expression in EM III/IV, individual marker comparisons between groups showed no significant differences (Table S5).

### 3.4 Ectopic Endometrial Epithelial sEVs and PF‐sEV Suppress Macrophage Phagocytosis

pMΦs play a critical role in immune homeostasis and tissue remodelling in response to injury within the peritoneal cavity (Haney 2000). In EM, pMΦ exhibits an altered transcriptomic profile (Henlon et al. 2024), impaired phagocytic capacity (Chuang et al. 2009; Zou et al. 2021; Wu et al. 2005; Liu et al. 2019) and a prorepair phenotype (Yonemura et al. 2024). Mouse studies have shown that pMΦ depletion promotes the establishment and growth of endometriotic implants (Haber et al. 2008) and enhances lesion vascularisation (Bacci et al. 2009).

Building on the distinct immunomodulatory profiles of ECT EEO‐sEVs and PF‐sEVs in EM III/IV, we hypothesised that lesion‐derived EVs regulate macrophage function. The shared profiles between PF‐sEVs and EEO‐sEVs suggest that EEO‐sEVs may diffuse into the peritoneal cavity, contributing to PF‐sEV composition, where they can interact with pMΦ. We hypothesised that by suppressing macrophage phagocytosis, these lesion‐derived sEVs contribute to immune evasion and lesion establishment in the peritoneal cavity.

**FIGURE 6:**
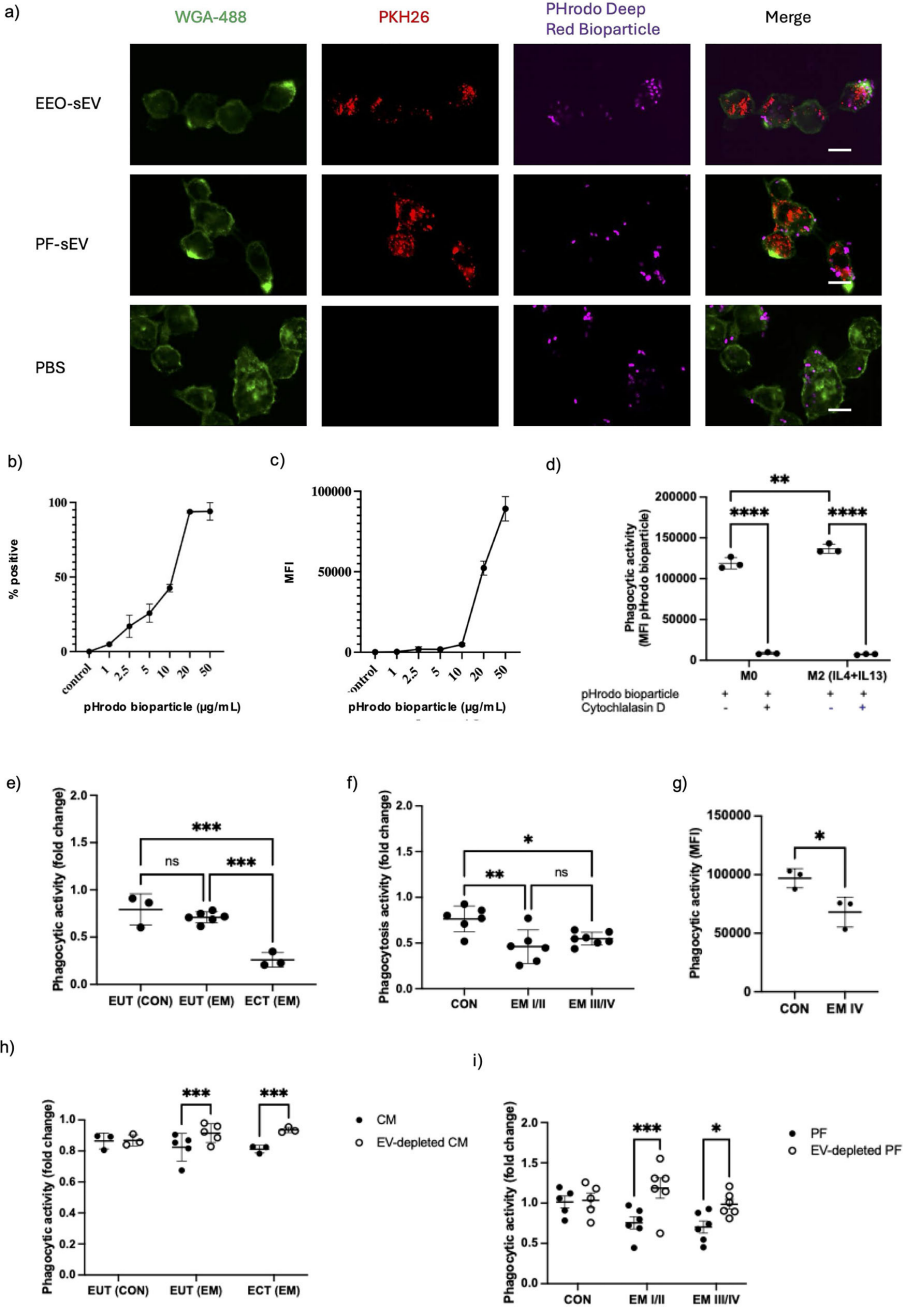
sEV suppresses phagocytic activity of macrophages in the peritoneal microenvironment in endometriosis. (a) Representative image of THP‐1‐derived macrophages were incubated with pHrodo *E. coli* deep red bioparticles (purple) in the presence (+) or absence (−) of PF‐sEV (PKH‐26 labelled, red) pretreatment for 24 h. Cell membranes were stained with WGA (green). Scale bar, 10 µm. (b, c) THP‐1‐derived macrophages were incubated with 1–50 µg/mL of pHrodo *E. coli* deep red bioparticles for 1 h. Phagocytic activity was assessed by flow cytometry (a) percentage of phagocytic THP‐1 derived macrophages compared to and (b) MFI at different bioparticle concentrations indicating overall phagocytic activity. Data are shown as mean nMFI ± SD (*n* = 3). (d) THP‐1‐derived M0 macrophages and IL‐4 + IL‐13 stimulated M2 macrophages were incubated with pHrodo *E. coli* deep red bioparticles (20 µg/mL, 1 h) with or without cytochalasin D pretreatment. Data are shown as mean nMFI ± SD (*n* = 3). **p <0.01, ****p < 0.0001. (e, f) Flow cytometry analysis of phagocytic activity in THP‐1‐derived macrophages treated with (e) EEO‐sEV from EUT (CON), EUT (EM) and ECT (EM) and (f) PF‐sEV from CON, EM I/II and EM III/IV for 24 h. Phagocytic activity was assessed using MFI of pHrodo *E. coli* deep red bioparticles and compared as a fold change. Fold change was calculated by dividing the deep red MFI of PF‐sEV‐treated cells by the mean deep red MFI of untreated cells from three wells. Data are presented as mean ± SD. *p < 0.05, **p < 0.01. (g) Flow cytometry analysis of phagocytic activity in pMΦ isolated from CON treated with PF‐sEVs from CON and EM patients (*n* = 3). Phagocytic activity was assessed using MFI of pHrodo *E. coli* deep red bioparticles. Data are presented as mean ± SD. *p < 0.05. (h, i) THP‐1‐derived macrophages were treated with (h) 5% (v/v) PF and EV‐depleted PF and (i) CM from 72 h incubation of EEO and EV‐depleted EEO for 24 h. Phagocytic activity was assessed using MFI of pHrodo *E. coli* deep red bioparticles and compared as a fold change. Data are presented as mean ± SD. *p < 0.05, ***p < 0.001. CM, culture media; CON, control; ECT, ectopic epithelium; EEO, endometrial epithelial organoid; EM, endometriosis; EUT, eutopic epithelium; MFI, median fluorescence intensity; PF, peritoneal fluid; pMΦ, peritoneal macrophage; sEV, small extracellular vesicle; WGA, wheat germ agglutinin.

Confocal microscopy confirmed the uptake of pHrodo bioparticles, EEO‐sEVs and PF‐sEVs (sEVs were prelabelled with membrane stain PKH26) by THP‐1‐derived M0 macrophages (Figure 6a). Cell membranes were stained with WGA‐488 to define cell boundaries, showing internalised fluorescence and no extracellular signal. Most cells displayed phagocytosis, while the extent of uptake was quantified by flow cytometry. We examined macrophage phagocytosis using a pHrodo bioparticle uptake assay, which demonstrated a clear dose–response in THP‐1‐derived macrophages (Figure 6b, c).

Macrophages in the peritoneal microenvironment have been found to exhibit prorepair characteristics and typically display high phagocytic activity (Bacci et al. 2009), which has been reported to be impaired in EM (Chuang et al. 2009; Zou et al. 2021; Wu et al. 2005; Liu et al. 2019), we focused on THP‐1‐derived M2 macrophages (IL‐4 + IL‐13 stimulated). Flow cytometry analysis confirmed increased expression of CD163 and CD206 following stimulation compared with M0 macrophages (Figure S6). M2 macrophages displayed a higher baseline phagocytic activity than M0 macrophages (*p* < 0.01) (Figure 6d). All cells showed a significant reduction in bioparticle uptake with cytochalasin D (*p* < 0.0001), confirming assay specificity.

To minimise the influence of media‐derived EVs, EEOs were cultured in EV‐depleted media for 72 h during EV collection. THP‐1‐derived M2 macrophages were treated with EEO‐sEVs from the three described sources for 24 h, after which phagocytic activity was assessed by flow cytometry (Figure 6e). Macrophages treated with ECT EEO‐sEVs showed significantly reduced phagocytic activity compared to those treated with EUT EEO‐sEVs from both EM patients and controls (*p* < 0.0001).

Similarly, treatment with PF‐sEVs from the EM Groups I/II and III/IV led to significantly reduced phagocytic activity in macrophages compared to the CON group (Figure 6f). Comparable results were observed in primary pMΦs treated with PF‐sEVs from EM and CON patients (Figure 6g). To further validate the suppressive effect of sEVs, we performed EV depletion on EEO CM and PF (validated by NTA; Figure S4). Phagocytic suppression was notably attenuated in macrophages treated with EV‐depleted EEO CM or PF (Figure 6h, i). Treatment with CM from ECT EEO cultures did not reduce macrophage phagocytic activity to the same extent as treatment with purified EEO‐sEVs. Purified EEO‐sEVs were isolated and concentrated from 5.5 mL of CM before treatment, whereas direct CM treatment exposed macrophages to a lower sEV concentration. Additionally, CM contains a mixture of soluble factors that may influence macrophage function independently of sEVs.

### 3.5 CD47/SIRP‐α Pathway Mediates sEV‐Induced Suppression of Macrophage Phagocytosis

To investigate the EV‐associated signalling pathway responsible for the suppression of phagocytic activity of macrophages in the peritoneal microenvironment, we examined the ‘don't eat me’ signal CD47. CD47 interacts with signal regulatory protein‐α (SIRP‐α) (Adams et al. 1998) to facilitate immune evasion and suppress macrophage phagocytosis in cancer, with evidence showing their regulation through sEVs (Li et al. 2022; Kaur et al. 2018). This pathway has been implicated in immune evasion in EM (Li et al. 2022), and recent studies have reported elevated CD47 expression in the ECT endometrium of EM patients (Li et al. 2022; Hu et al. 2022). Furthermore, pMΦs isolated from women with EM exhibit increased expression of SIRP‐α compared to patients without EM (Xie et al. 2019).

**FIGURE 7:**
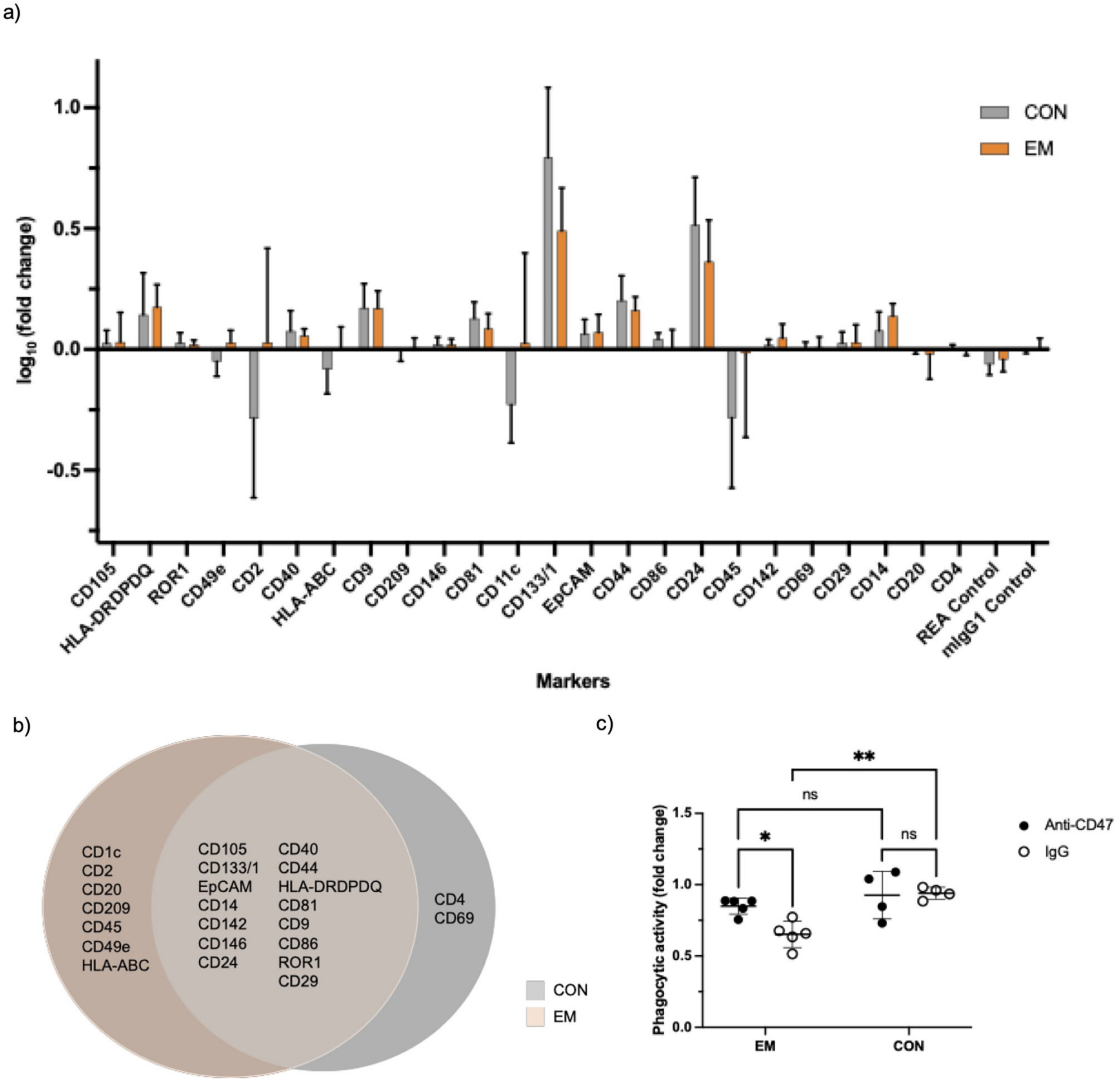
sEV mediates phagocytic suppression via CD47 in endometriosis. (a) PF‐sEVs from control (*n* = 5) and women with endometriosis (*n* = 5) were analysed for CD47 coexpression using the MACSPlex EV Kit IO. The data were normalised by fold change, calculated as the ratio of MFI from EV‐positive beads to that of PBS‐only negative control beads for each marker. (b) The Venn diagram of coexpressed markers with CD47 on PF‐sEV between CON (*n* = 5) and EM (*n* = 5). (c) Flow cytometry analysis of the effect of blocking CD47 on PF‐sEV on regulating macrophage phagocytosis. THP‐1‐derived macrophages were treated with PF‐sEV from EM or CON, preincubated with either IgG (control) or anti‐CD47 antibodies. Phagocytic activity was assessed using pHrodo *E. coli* deep red bioparticles. Fold change was calculated by dividing the deep red MFI of PF‐sEV‐treated cells by the mean deep red MFI of untreated cells from three wells. Data are shown as mean ± SD. *p < 0.05, **p < 0.01. CON, control; ECT, ectopic epithelium; EEO, endometrial epithelial organoid; EM, endometriosis; EUT, eutopic epithelium; MFI, median fluorescence intensity; PF, peritoneal fluid; sEV, small extracellular vesicle.

We assessed the coexpression of CD47 with known EV markers on PF‐sEVs from control (*n* = 5) and EM patients (*n* = 5) using a modified MACSPlex EV kit protocol (Figure 7a). Patient clinical characteristics and EV characterisation by NTA are provided in Table S6. Out of 37 markers examined on PF‐sEVs, 15 were found in both control and EM patients based on a threshold of ≥3 positive detections (a linear fold change greater than 1) out of five samples per group (Figure 7b). CD81, CD9, CD133/1, EpCAM, CD24, HLA‐DRDPDQ and CD14 are expressed in all 10 samples, indicating CD47 expression by both epithelium and macrophage‐derived sEVs.

To test the role of CD47 in regulating macrophage phagocytosis, we disrupted CD47 on the surface of PF‐sEVs using a blocking anti‐CD47 monoclonal antibody. THP‐1‐derived M2 macrophages treated with anti‐CD47 PF‐sEVs from EM patients showed elevated phagocytic activity compared to the isotype control IgG‐treated PF‐sEVs group (Figure 7c). The phagocytic activity of macrophages treated with anti‐CD47 PF‐sEVs from EM approached levels similar to those treated with IgG PF‐sEVs from CONs. This suggests that CD47 blockade could potentially normalise the impaired phagocytic function associated with EM. Supporting the importance of this pathway, we confirmed high SIRP‐α expression in pMΦ from EM patients (*n* = 3) (Figure S7), as previously reported (Xie et al. 2019), highlighting the CD47/SIRP‐α axis as a key mediator of macrophage function in EM pathophysiology.

## 4 Discussion

In this study, we aimed to investigate the cellular origins and functional characteristics of sEVs within the peritoneal microenvironment, with a particular focus on those derived from EM lesions. To our knowledge, this is the first study to characterise sEVs from lesion‐originated EE cell using an EEO model, a validated model to capture lesion transcriptomic characteristics in vitro (Boretto et al. 2019).

We observed variability in EEO growth at P0, likely due to differences in dissociation methods and starting tissue types. Lesion tissues required more prolonged enzymatic digestion and generally yielded fewer epithelial cells than EUT endometrial biopsies. Additionally, limited EEO growth from EUT biopsies obtained from individuals receiving HT is consistent with reports describing HT‐associated endometrial thinning and atrophy (Bastianelli et al. 2020). However, subsequent passages (P3–P5) showed consistent growth and epithelial marker expression, indicating that the EEO model is stable and suitable for comparative sEV analysis.

Surface profiling of EEO‐sEVs revealed high expression of epithelial markers EpCAM, CD133/1, along with the immunomodulatory marker CD24. Epithelial cell‐derived sEVs have been reported to modulate the immune response by interacting with immune cells in the lung (Moon et al. 2015) and intestinal tract (Jiang et al. 2016). Notably, lesion‐derived EEO‐sEVs exhibited elevated expression of CD44 and CD29, with relatively lower EpCAM expression when compared to EEO‐sEVs from EUT endometrium. CD44, a cell‐surface glycoprotein, is involved in cell–cell interactions, cell adhesion and migration (Ponta et al. 2003). By interacting with peritoneal mesothelial cell (PMC)‐associated hyaluronan, CD44 in cancer cells [ovarian (Lessan et al. 1999) and gastric cancer (Nishimura et al. 1996)] and endometrial cells (Dechaud et al. 2001) has been found to responsible for the attachment to mesothelium. Decreased development of endometriotic lesions was observed in CD44 knockout mice (Knudtson et al. 2015). Soluble CD44 is high in PF of EM (Hasegawa et al. 2008; Mashayekhi et al. 2015). CD29, also known as integrin β1, plays a role in cell adhesion and signalling (Fransvea et al. 2009) and contributes to ovarian cancer cell adhesion to mesothelium (Lessan et al. 1999).

This shift in marker expression is consistent with epithelial‐mesenchymal transition (EMT), a biological process in which epithelial cells lose polarity and cell–cell adhesion, acquiring mesenchymal characteristics including increased motility. This transformation has been linked to the progression of EM (Geng et al. 2013), and downregulation of EpCAM is a recognised feature of this transition (Sankpal et al. 2017). Furthermore, the high expression of CD44 and CD29 has been linked to EMT (Geng et al. 2013).

It is well established that the content of EV, including their protein and surface marker composition, is highly dependent on the source cells (Villarroya‐Beltri et al. 2014). The sEV surface profile mirrors these cellular changes, further supporting the notion that endometriotic lesions exhibit a more invasive and mesenchymal phenotype (Yang and Yang 2017; Matsuzaki and Darcha 2012).

Furthermore, this is the first study to apply a multiplex bead‐based assay to examine the cellular origins of PF derived sEVs (PF‐sEVs). The technique has been applied to serum/plasma derived EV profiling in haematological malignancies (Li et al. 2022) and Parkinson disease (Vacchi et al. 2020). Our findings indicate multiple cellular sources, including EE cells (EpCAM, CD133/1), immune cells (macrophages: CD14; T cells: CD3; B cells: CD25), as well as potential contributions from endothelial cells (CD105, CD146) and platelets (CD42a). High EpCAM levels in PF‐sEVs from CON individuals suggest contributions from EUT endometrium, potentially via retrograde menstruation (Sampson 1927). Marker correlation analysis further supports enrichment patterns consistent with distinct cell origins.

Comparative in vitro analyses of sEVs derived from EEO and pMΦs revealed distinct surface marker signatures, consistent with their respective cellular phenotypes. EEO‐derived sEVs prominently expressed epithelial‐specific surface markers, including EpCAM and CD133/1, whereas sEVs derived from pMΦs expressed immune‐associated markers such as CD14 and CD45.

PCA of surface marker expression profiles revealed distinct clustering of PF‐sEVs of individuals with advanced‐stage EM (EM III/IV), compared to early‐stage (EM I/II) and control (CON) groups. Notably, EM III/IV group exhibited enhanced expression of immune‐related markers, including CD40, CD8, HLA‐DRDPDQ, CD4, CD44 and CD86. This observation aligns with previous findings that soluble CD44 levels are significantly elevated in the PF of patients with Stage III/IV EM (Hasegawa et al. 2008), and with reports of dysregulated T cell and macrophage populations contributing to disease pathogenesis.

Although PCA and unsupervised clustering identified group‐level distinctions, analysis of individual marker expression did not find statistically significant differences. This underscores the complexity of intercellular communication and marker coexpression in the peritoneal niche. These findings support the utility of multivariate and unsupervised methods to uncover subtle, yet biologically relevant alterations associated with EM progression.

Finally, we focused on the role of PF‐sEV, especially lesion derived sEVs on phagocytic activity of pMΦ, an important mechanism in immune surveillance. Recruitment and trafficking of pMΦs during injury and tumour metastasis have been consistently observed in various visceral organs (Hossain et al. 2022; Honda et al. 2021). In EM, pMΦ is the dominant immune population in the peritoneal microenvironment and represents one of the three major sources of lesion‐infiltrated macrophages (Hogg et al. 2021). Impaired phagocytosis by pMΦ has been described in EM (Chuang et al. 2009; Zou et al. 2021; Wu et al. 2005; Liu et al. 2019).

Using *pHrodo* bioparticles—pH‐sensitive fluorescent reporters that selectively emit fluorescence upon internalisation into the acidic environment of phagolysosomes—we assessed macrophage phagocytic activity by flow cytometry. This technique enables real‐time, kinetic monitoring of phagocytosis and is highly specific, as fluorescence only occurs in low pH environments, thereby significantly reducing background signal and false positives (Lindner et al. 2020). It is compatible with both confocal microscopy and flow cytometry for quantitative and spatial analysis of phagocytic function (Lindner et al. 2020; Neaga et al. 2013).

We observed that PF‐sEVs isolated from EM patients suppressed the phagocytic activity of THP‐1‐derived M2 macrophages. In contrast, this effect was not observed in macrophages treated with PF‐sEVs from CON. A similar suppression was observed in primary pMΦ isolated from a healthy control donor. Importantly, blocking surface CD47 on PF‐sEVs using a neutralising antibody restored phagocytic activity, implicating the CD47/SIRP‐α axis—a known ‘don't eat me’ signalling pathway—in sEV‐mediated immune suppression in EM. Coexpression of CD47 with epithelial markers EpCAM, CD133/1 and the immunomodulatory molecule CD24 on PF‐sEVs further supports the hypothesis that lesion‐derived epithelial sEVs contribute to immune evasion by modulating macrophage activity within the peritoneal microenvironment. When conditioned medium was used instead of isolated sEVs, the inhibitory effect on phagocytosis was less pronounced. Although the sEV concentrations used were within the physiological range estimated from NTA data of PF, in vivo these sEVs exist within a multifactorial extracellular environment rich in soluble factors and other EV populations that can influence macrophage responses. These in vitro findings suggest a phagocytic suppressive effect of lesion‐derived sEVs; however, future patient‐matched or in vivo studies will be needed to confirm whether such interactions occur under physiological conditions.

Several limitations of the current study should be acknowledged. The absence of CD10, a known ES cell marker, represents a significant gap in the characterisation of this large and important cell population in EM lesions and EUT endometrium (Konrad et al. 2018). Additionally, while the MACSPlex technique provides valuable semi‐quantitative data on EV surface markers, it does not allow for precise quantification of marker proportions within the whole EV population. This limitation highlights the potential benefit of complementary single EV flow cytometry approaches in future studies to provide a more detailed and quantitative characterisation of EV subpopulations.

Our findings establish the EEO model as a robust and tractable in vitro platform for studying EV biology in EM. EEO models have also been proven to retain hormonal responsiveness, responding to sequential oestrogen and progesterone treatment with morphologic and transcriptomic changes reflective of in vivo endometrium, including expression of *PAEP* and *SPP1* (Boretto et al. 2017; Turco et al. 2017). Recent findings demonstrate lesion‐specific hormonal responsiveness in ECT EEO, with reduced sensitivity to oestrogen and progesterone compared to EUT EEO (Zhang et al. 2025). Given the hormone dependence of EM and aberrant oestrogen accumulation in ECT lesions (Zeitoun and Bulun 1999), accurately modelling pathological states requires mimicking both the differential hormone levels and tissue‐specific responses in culture. Hormones play a crucial role in shaping EV profiles. The hormonal fluctuation during the menstrual cycle (Toth et al. 2007) and menopause (Rank et al. 2012) induces changes in EV profiles. In deep infiltrating EM, one study has revealed that plasma‐derived large EVs decrease in women using combined oral contraceptive pills (Carrillo Torres et al. 2023). Incorporating these hormonal dynamics into EEO will be crucial for identifying disease‐relevant sEV signatures reflective of the peritoneal microenvironment.

Importantly, the EEO model also serves as an effective system for assessing epithelial‐specific expression profiles of lesion‐derived cells, in contrast to bulk lesion tissue, which is predominantly stromal in composition. Future research should aim to correlate EV surface signatures with matched cellular expression data to better delineate the source and functional relevance of specific sEV phenotypes. Furthermore, utilisation of organoid models that incorporate stromal cell coculture could also be used to recapitulate the in vivo lesion EV secretome.

Finally, in addition to the observation that lesion‐derived sEVs carry immunomodulatory molecules such as CD47 and adhesion‐related markers including CD44 and CD29, previous studies in cancer have demonstrated that EVs can mediate the intercellular transfer of surface receptors, thereby influencing recipient cell behaviour (Languino et al. 2016). For example, beyond direct interaction with inhibitory receptors, ‘don't eat me’ signals like PD‐L1 have been shown to transfer to the membrane of PD‐L1‐negative breast cancer cells via EV, facilitating immune evasion (Yang et al. 2018). Future studies should explore this possibility and determine whether EV‐mediated receptor transfer of CD47, CD44 and CD29 contributes to lesion migration, immune escape and peritoneal invasion. Furthermore, it will be important to assess whether lesion‐derived sEVs influence macrophage activation and polarisation in addition to their effects on phagocytic function, as such immunomodulatory mechanisms may play a broader role in shaping the peritoneal microenvironment.

## Author Contributions

**Yifan Wang**: conceptualisation, writing – original draft, investigation, methodology. **Zhixing Jin**: methodology, investigation. **Abigail Freeman Blatchford**: methodology, investigation. **Banayot Hosh**, **Malak Amer** and **Ayazhan Akhatova**: investigation. **Krina Zondervan**: funding acquisition, supervision. **Erin Greaves**: supervision, funding acquisition. **Rebecca Dragovic**: conceptualisation, writing – review and editing, formal analysis, supervision. Christian M.Becker: supervision, funding acquisition, conceptualisation, resources. **Jen Southcombe**: supervision, writing – review and editing, funding acquisition, conceptualisation, resources.

## Funding

This study was funded by the Nuffield Department of Women's and Reproductive Health, University of Oxford, and also supported by the University of Oxford Medical Sciences Internal Fund Award [0014469], Academy of Medical Science Award [SBF007∖100078] and Medical Research Council Award [MR/W028255/1].

## Conflicts of Interest

Christian M. Becker and Krina Zondervan report that in the past 3 years they received funding supporting research (funds to the Institution) from Aspira Labs, Inc., Bayer AG, Chemo Research S.L., Proteomics International PTY LTD, Roche Diagnostics GmbH, unrelated to the content presented in the paper. Christian M. Becker has a consultancy role with ObsEva, Theramex, Roche Diagnostics, Sumitovant, Gedeon Richter and Research Grants from Bayer and Serac Life Services. Krina Zondervan is a board member (non‐remunerated) of the World Endometriosis Research Foundation. The remaining authors declare that the research was conducted in the absence of any commercial or financial relationships that could be construed as a potential conflicts of interest.

## Supporting information




**Supplementary Figures**: jev270227‐sup‐0001‐Figures.pptx

**Supplementary Tables**: jev270227‐sup‐0002‐Tables.docx

## Data Availability

The data that support the findings of this study are available from the corresponding author upon reasonable request.
