# Supplementary material for: Human Endometriotic Lesion‐Derived Small Extracellular Vesicles Impair Macrophage Function in the Peritoneal Microenvironment
Source: J Extracell Vesicles. 2026 Feb 19;15(2):e70227. doi: 10.1002/jev2.70227 (PMC12919373; doi:10.1002/jev2.70227)
Supplement: Supplementary file 1 — Supplementary Figures: jev270227‐sup‐0001‐Figures.pptx [file JEV2-15-e70227-s001.pptx]

## Slide 1
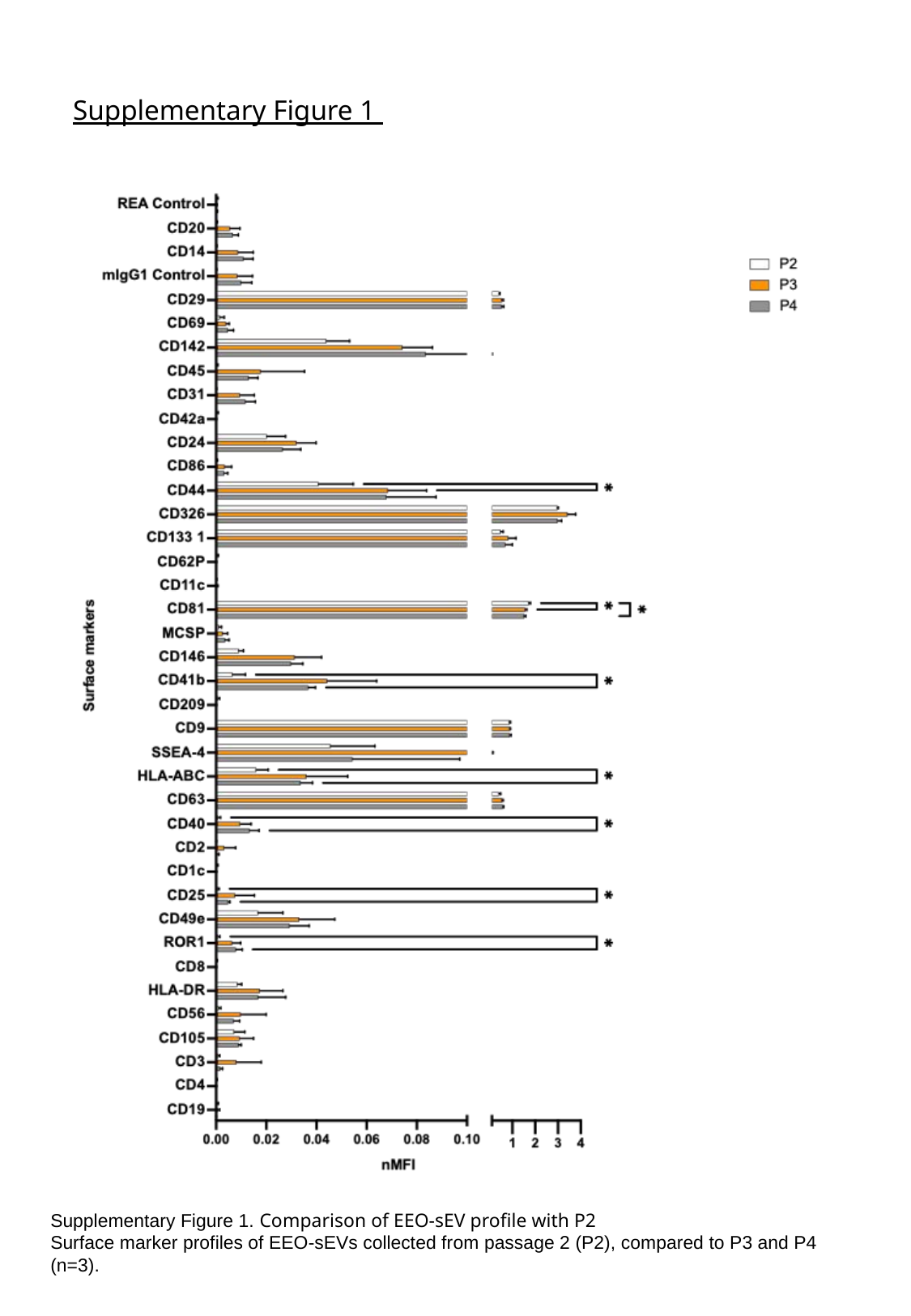

Supplementary Figure 1
Supplementary Figure 1. Comparison of EEO-sEV profile with P2
Surface marker profiles of EEO-sEVs collected from passage 2 (P2), compared to P3 and P4 (n=3).

## Slide 2
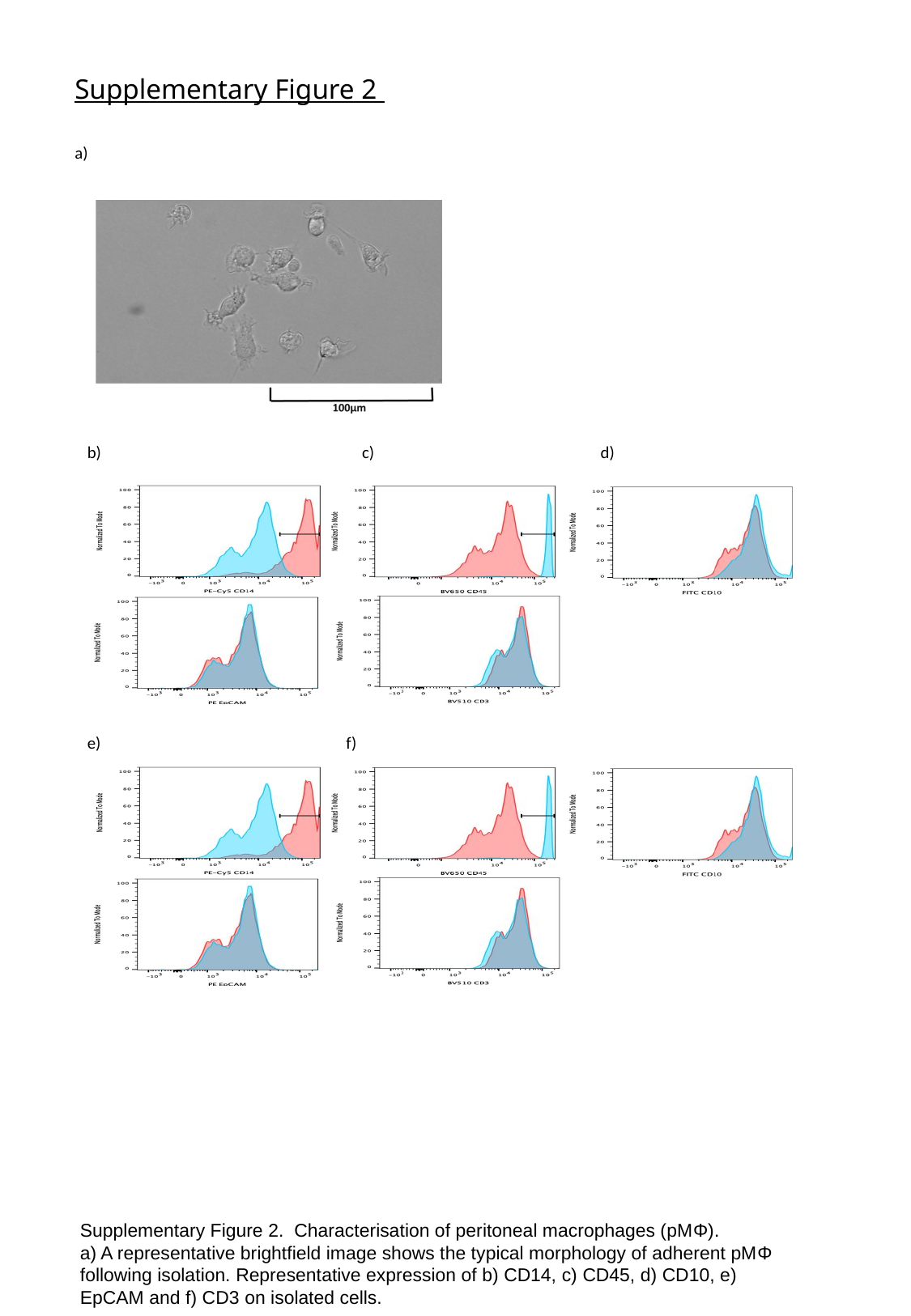

# Supplementary Figure 2
a)
b) c) d)
e) f)
Supplementary Figure 2. Characterisation of peritoneal macrophages (pMΦ).
a) A representative brightfield image shows the typical morphology of adherent pMΦ following isolation. Representative expression of b) CD14, c) CD45, d) CD10, e) EpCAM and f) CD3 on isolated cells.

## Slide 3
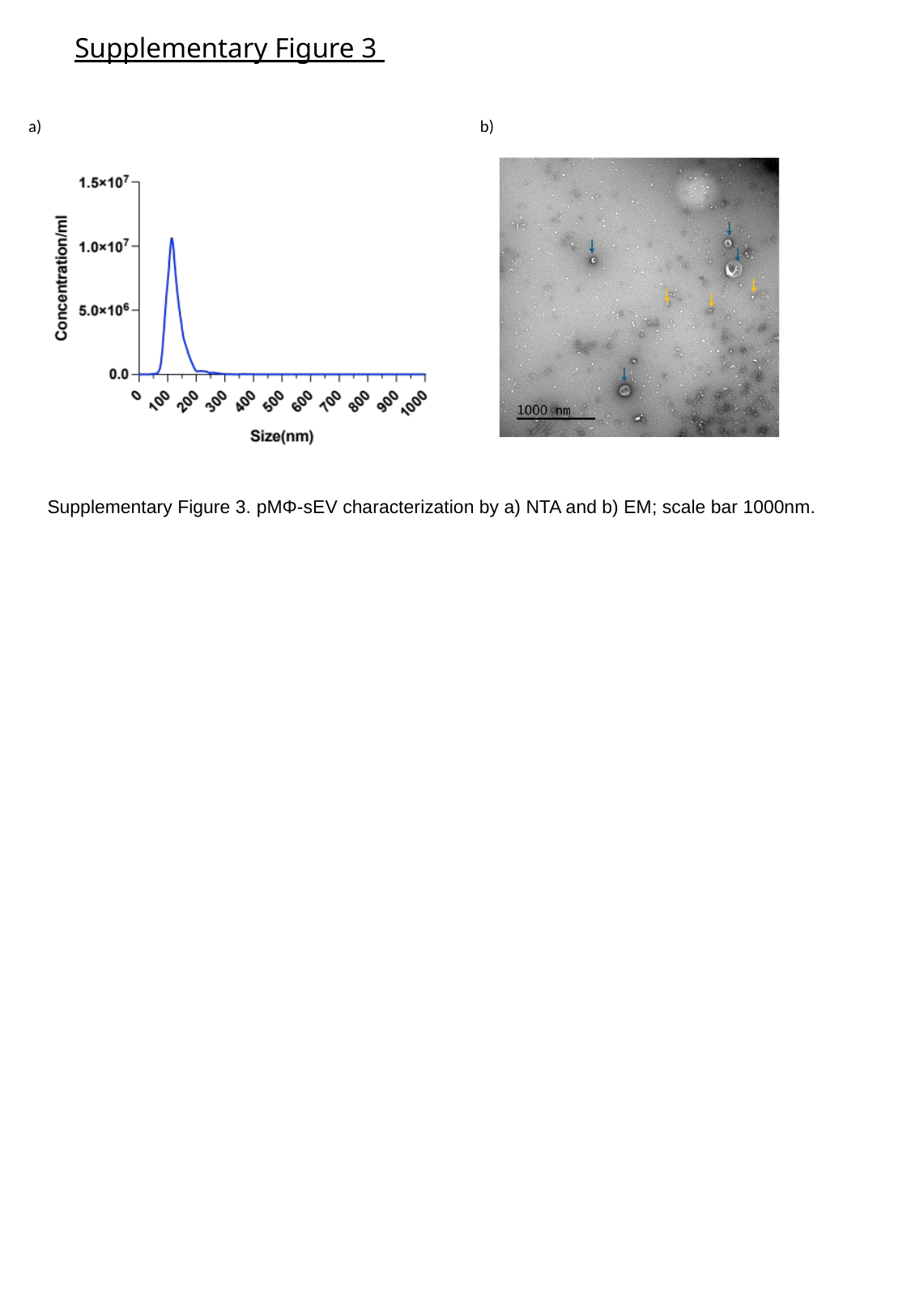

Supplementary Figure 3
a)                                                                                                                  b)
Supplementary Figure 3. pMΦ-sEV characterization by a) NTA and b) EM; scale bar 1000nm.

## Slide 4
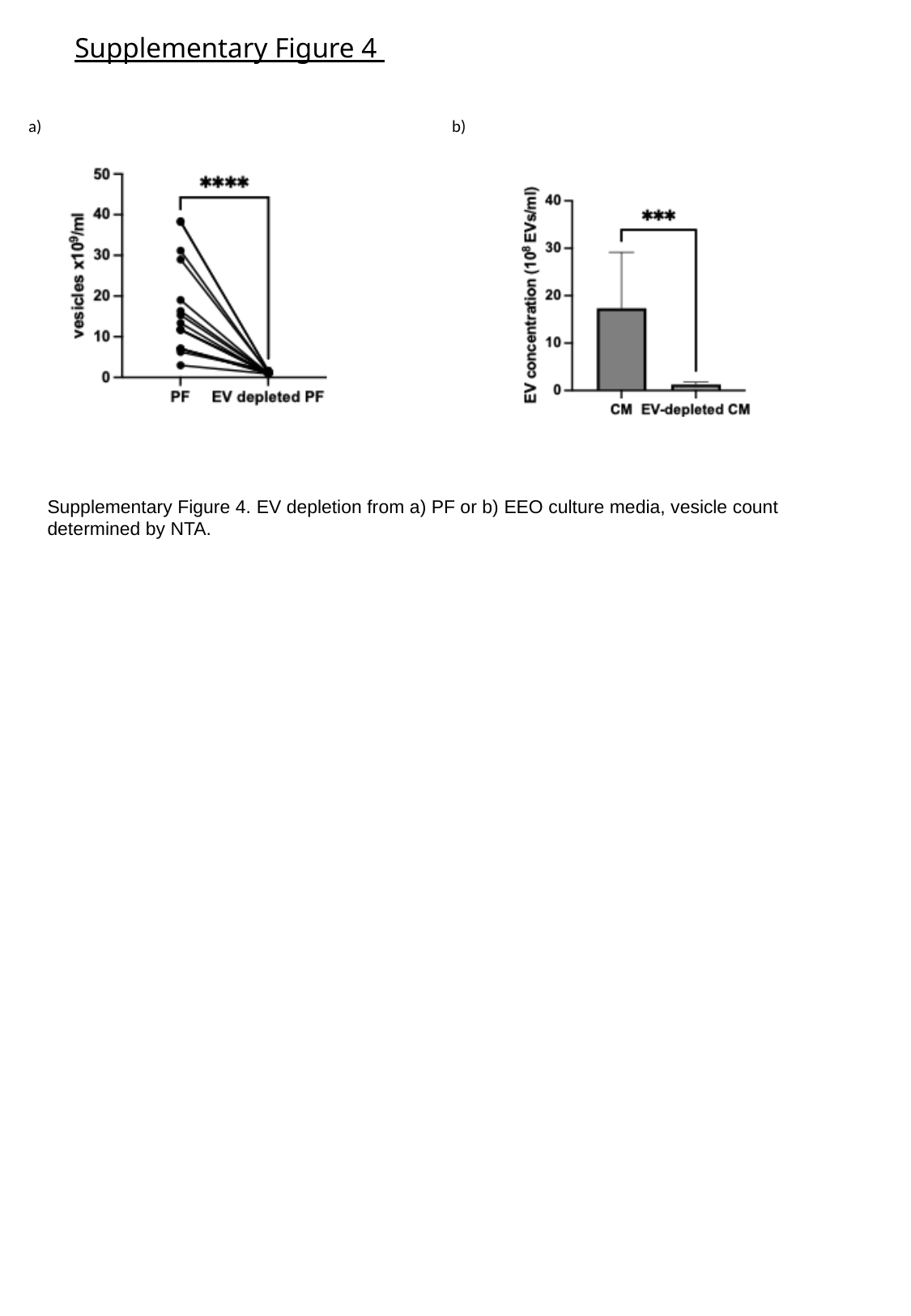

Supplementary Figure 4
a) b)
Supplementary Figure 4. EV depletion from a) PF or b) EEO culture media, vesicle count determined by NTA.

## Slide 5
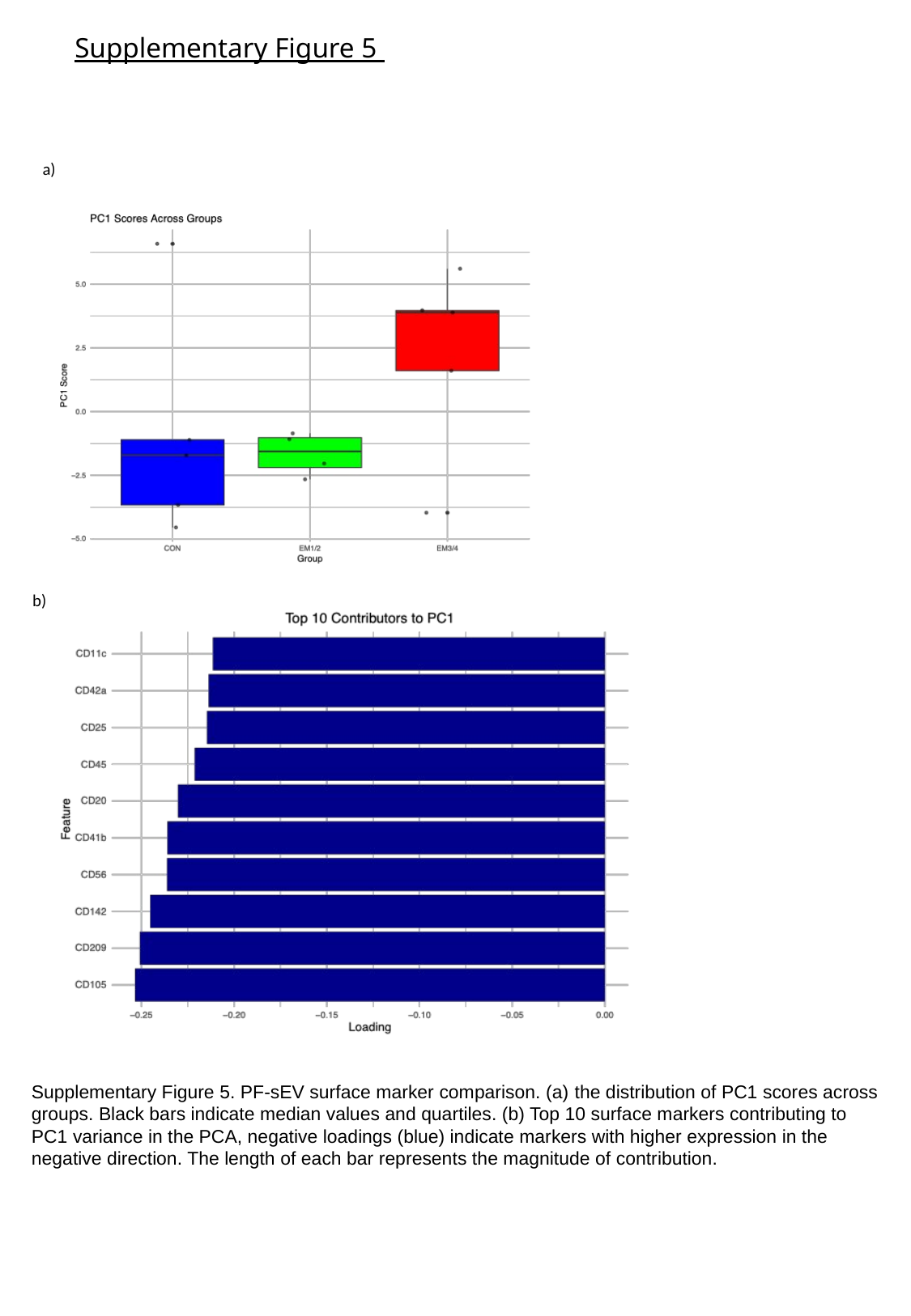

Supplementary Figure 5
a)
b)
Supplementary Figure 5. PF-sEV surface marker comparison. (a) the distribution of PC1 scores across groups. Black bars indicate median values and quartiles. (b) Top 10 surface markers contributing to PC1 variance in the PCA, negative loadings (blue) indicate markers with higher expression in the negative direction. The length of each bar represents the magnitude of contribution.

## Slide 6
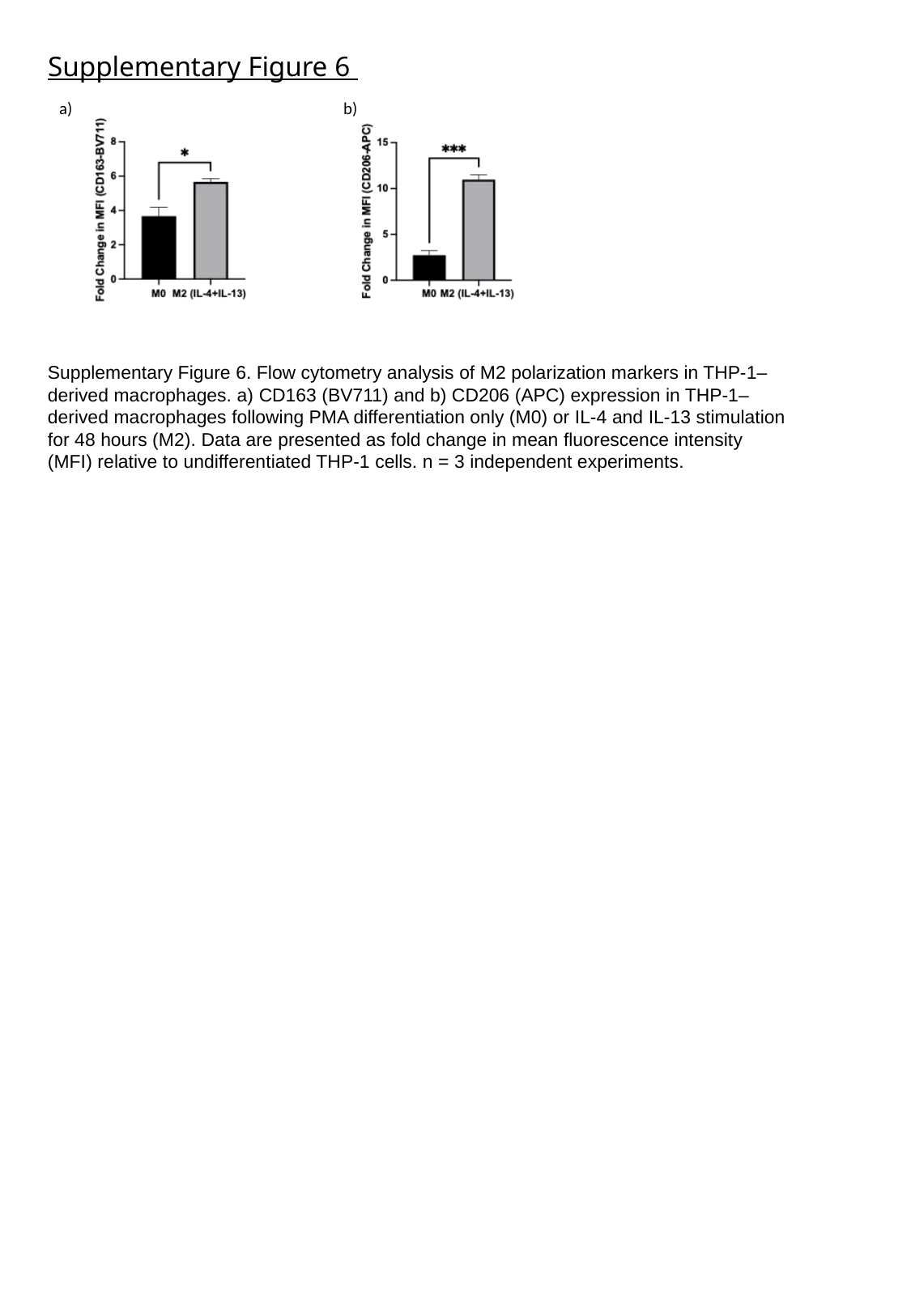

Supplementary Figure 6
                                                                  b)
Supplementary Figure 6. Flow cytometry analysis of M2 polarization markers in THP-1–derived macrophages. a) CD163 (BV711) and b) CD206 (APC) expression in THP-1–derived macrophages following PMA differentiation only (M0) or IL-4 and IL-13 stimulation for 48 hours (M2). Data are presented as fold change in mean fluorescence intensity (MFI) relative to undifferentiated THP-1 cells. n = 3 independent experiments.

## Slide 7
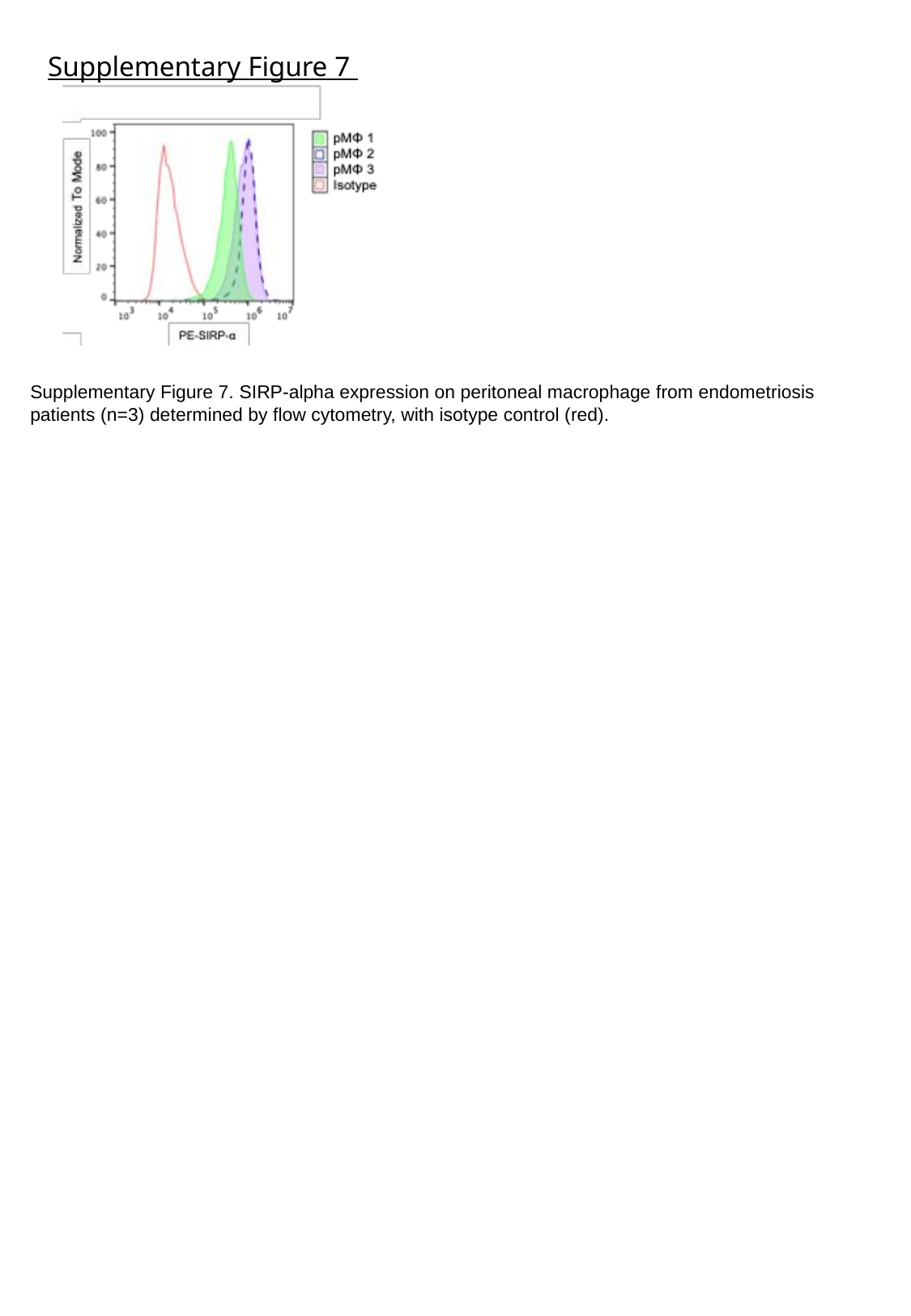

Supplementary Figure 7
Supplementary Figure 7. SIRP-alpha expression on peritoneal macrophage from endometriosis patients (n=3) determined by flow cytometry, with isotype control (red).
