# Supplementary material for: Human Endometriotic Lesion‐Derived Small Extracellular Vesicles Impair Macrophage Function in the Peritoneal Microenvironment
Source: J Extracell Vesicles. 2026 Feb 19;15(2):e70227. doi: 10.1002/jev2.70227 (PMC12919373; doi:10.1002/jev2.70227)
Supplement: Supplementary file 2 — Supplementary Tables: jev270227‐sup‐0002‐Tables.docx [file JEV2-15-e70227-s002.docx]

**Supplementary tables**

**Table S1. Expansion media (ExM) for EEO culturing.** The table provides information on each product, the company that supplied it, and its final concentration in the media.

| Product | Company | Final Concentration |
| --- | --- | --- |
| Advanced DMEM/F12 | Thermo Fisher | 1X |
| B27 Supplement minus Vitamin-A | Thermo Fisher | 1X |
| Recombinant human Noggin | Peprotech | 100ng/mL |
| Recombinant human FGF-10 | Peprotech | 10ng/mL |
| N2 Supplement (100X) | Thermo Fisher | 1X |
| N-Acetyl-cysteine | Sigma Aldrich | 1.25mM |
| Primocin | InvivoGen | 100µg/mL |
| Recombinant human Rspondin-1 | Peprotech | 500ng/mL |
| Nicotinamide | Sigma Aldrich | 2mM |
| Recombinant human EGF | Peprotech | 50ng/mL |
| ALK-4,-5,-7 Inhibitor, A83-01 | Tocris | 500nM |
| Insulin-Transferrin-Selenium (100X) | Thermo Fisher | 1X |
| bEGF | Peprotech | 2ng/ml |
| SB202190 | Sigma Aldrich | 10uM |
| GlutaMax (100X) | Thermo Fisher | 1X |
| β-Estradiol | Sigma Aldrich | 1nM |

**Table S2. Primary and secondary antibodies used for immunofluorescence and Western blot analyses.** IF, Immunofluorescence; WB, Western blot; N/A, not applicable

| **Target Protein** | **Antibody** | **Clone** | **Host** | **Dilution** | **Use** | **Source** |
| --- | --- | --- | --- | --- | --- | --- |
| E-Cadherin | E-cadherin conjugated-Alexa 647 | DECMA-1 | Mouse | 10 µg/mL | IF | BioLegend, USA |
| Pan-Cytokeratin | Pan-cytokeratin conjugated-Alexa 488 | AE1/AE3 | Mouse | 1 µg/mL | IF | Invitrogen, USA |
| ZO-1 | Anti-ZO-1 (primary) | ZO1-1A12 | Mouse | 10 µg/mL | IF | Invitrogen, USA |
| Laminin | Anti-Laminin (primary) | Polyclonal | Rabbit | 20 µg/mL | IF | Invitrogen, USA |
| EpCAM | Anti-EpCAM (primary) | VU1D9 | Mouse | 4 µg/mL | IF | Invitrogen, USA |
| DAPI | Mounting Medium with DAPI - Aqueous, Fluoroshield | N/A | N/A | N/A | IF | Abcam, UK |
| Secondary (IF) | Goat anti-Rabbit IgG (H+L) Cross-Adsorbed Secondary Antibody, Alexa Fluor 594 | Polyclonal | Goat | 2 µg/mL | IF | Invitrogen, USA |
| Secondary (IF) | Goat anti-Mouse IgG (H+L) Cross-Adsorbed Secondary Antibody, Alexa Fluor 488 | Polyclonal | Goat | 1 µg/mL | IF | Invitrogen, USA |
| Syntenin | Anti-Syntenin | EPR8102 | Rabbit | 1:1000 | Western Blot | Abcam, UK |
| Albumin | Anti-Albumin | Polyclonal | Rabbit | 1:5000 | Western Blot | Cell Signaling Technology, USA |
| Argonaute-1 | Anti-Argonaute-1 | D84G10 | Rabbit | 1:1000 | Western Blot | Cell Signaling Technology, USA |
| Secondary (WB) | HRP-conjugated swine anti-rabbit IgG | Polyclonal | Swine | 1:2000 | Western Blot | Dako, USA |

**Table S3.** **Surface marker expression on EEO-sEVs measured by multiplex bead-based flow cytometry excluded from quantitative analysis.** The expression level of each marker was measured as the normalised median fluorescence intensity (nMFI) of CD9⁺, CD63⁺, and CD81⁺ small extracellular vesicles (sEVs).

|  | EUT (CON) | | EUT (EM) | | ECT (EM) | |
| --- | --- | --- | --- | --- | --- | --- |
|  | Mean | SD | Mean | SD | Mean | SD |
| CD19 | 0.0004 | 0.0004 | 0.0001 | 0.0002 | 0.0003 | 0.0005 |
| CD4 | 0.0002 | 0.0004 | 0.0000 | 0.0002 | -0.0001 | 0.0005 |
| CD8 | 0.0003 | 0.0003 | 0.0001 | 0.0002 | 0.0000 | 0.0003 |
| CD1c | 0.0004 | 0.0004 | 0.0001 | 0.0002 | 0.0000 | 0.0003 |
| CD2 | 0.0002 | 0.0004 | 0.0004 | 0.0003 | 0.0001 | 0.0007 |
| CD209 | 0.0007 | 0.0003 | 0.0003 | 0.0002 | 0.0003 | 0.0003 |
| MCSP | 0.0000 | 0.0002 | -0.0001 | 0.0002 | -0.0003 | 0.0005 |
| CD11c | 0.0004 | 0.0004 | 0.0002 | 0.0002 | -0.0001 | 0.0005 |
| CD62P | 0.0003 | 0.0003 | 0.0002 | 0.0001 | -0.0001 | 0.0003 |
| CD86 | 0.0006 | 0.0004 | 0.0004 | 0.0002 | 0.0001 | 0.0005 |
| CD42a | 0.0013 | 0.0003 | 0.0007 | 0.0003 | 0.0005 | 0.0004 |
| CD31 | 0.0007 | 0.0002 | 0.0005 | 0.0002 | 0.0003 | 0.0006 |
| CD45 | 0.0013 | 0.0007 | 0.0016 | 0.0005 | 0.0018 | 0.0009 |
| CD14 | 0.0003 | 0.0002 | 0.0000 | 0.0002 | 0.0000 | 0.0005 |
| CD20 | 0.0006 | 0.0001 | 0.0001 | 0.0001 | 0.0002 | 0.0008 |

**Table S4. Surface marker profile of pMΦ- sEV measured by MACSPLEX EV kit IO.** Data are presented as mean ± SD. n=19.

| Antibody | Mean nMFI ± SD | Antibody | Mean nMFI ± SD |
| --- | --- | --- | --- |
| REA control | 0.003 ± 0.004 | **CD24** | 0.370 ± 0.248 |
| HLA-DRDPDQ | 4.306 ± 2.297 | **CD86** | 0.028 ± 0.013 |
| CD56 | 0.039 ± 0.017 | **CD44** | 0.743 ± 0.257 |
| CD105 | 0.127 ± 0.061 | **CD29** | 0.286 ± 0.209 |
| CD49E | 0.017 ± 0.009 | **CD142** | 0.013 ± 0.009 |
| SSEA-4 | 0.008 ± 0.004 | **CD45** | 0.126 ± 0.087 |
| HLA-ABC | 0.191 ± 0.100 | **CD31** | 0.022 ± 0.013 |
| CD81 | 1.530 ± 0.144 | **CD14** | 0.214 ± 0.135 |
| CD41B | 0.026 ± 0.017 | **CD9** | 0.930 ± 0.179 |
| mlgG1 control | 0.004 ± 0.004 | **CD63** | 0.581 ± 0.144 |
| CD3 | 0.065 ± 0.035 | **CD40** | 0.109 ± 0.105 |
| CD4 | 0.043 ± 0.026 | **CD11C** | 0.118 ± 0.078 |
| CD8 | 0.031 ± 0.035 | **MCSP** | 0.024 ± 0.013 |
| CD2 | 0.035 ± 0.044 | **ROR1** | 0.026 ± 0.031 |
| CD25 | 0.020 ± 0.013 |  |  |

**Table S5.** **Expression levels of surface markers on PF-sEVs from CON and EM (I/II and III/IV) patients as measured by** **multiplex bead-based flow cytometry.** nMFI (mean and SD) are presented for each group. Statistical significance was determined using Kruskal-Wallis test followed by Dunn's post-hoc test. PF, peritoneal fluid; CON, control; EM, endometriosis; nMFI, normalised median fluorescent intensity.

|  | CON | | EM I/II | | EM III/IV | | Group difference | |  |
| --- | --- | --- | --- | --- | --- | --- | --- | --- | --- |
|  |  |  |  |  |  |  |  |  |  |
|  | Mean | SD | Mean | SD | Mean | SD | p value | FDR |  |
| CD3 | 0.063 | 0.147 | 0.022 | 0.078 | -0.113 | 0.098 | 0.077 | 0.292 |  |
| CD4 | 0.003 | 0.034 | 0.018 | 0.006 | 0.009 | 0.017 | 0.406 | 0.612 |  |
| CD19 | 0.005 | 0.007 | 0.015 | 0.010 | -0.002 | 0.011 | 0.198 | 0.560 |  |
| CD8 | 0.054 | 0.036 | 0.085 | 0.057 | 0.141 | 0.094 | 0.316 | 0.596 |  |
| HLA-DRDPDQ | 0.230 | 0.119 | 0.354 | 0.138 | 0.694 | 0.558 | 0.161 | 0.545 |  |
| CD56 | 0.072 | 0.162 | 0.112 | 0.071 | -0.137 | 0.134 | 0.058 | 0.545 |  |
| CD105 | 0.009 | 0.039 | 0.012 | 0.003 | -0.009 | 0.022 | 0.304 | 0.596 |  |
| CD2 | 0.036 | 0.050 | 0.007 | 0.011 | 0.026 | 0.036 | 0.770 | 0.793 |  |
| CD25 | 0.035 | 0.059 | 0.027 | 0.007 | -0.049 | 0.050 | 0.086 | 0.545 |  |
| CD49e | 0.014 | 0.013 | 0.013 | 0.005 | -0.012 | 0.027 | 0.406 | 0.612 |  |
| ROR1 | 0.017 | 0.014 | 0.025 | 0.003 | 0.031 | 0.026 | 0.445 | 0.612 |  |
| CD9 | 0.912 | 0.104 | 0.787 | 0.153 | 0.718 | 0.094 | 0.062 | 0.545 |  |
| SSEA-4 | 0.025 | 0.015 | 0.012 | 0.009 | -0.010 | 0.032 | 0.273 | 0.579 |  |
| HLA-ABC | 0.117 | 0.103 | 0.134 | 0.023 | 0.061 | 0.145 | 0.510 | 0.612 |  |
| CD63 | 0.431 | 0.213 | 0.556 | 0.244 | 0.523 | 0.119 | 0.440 | 0.612 |  |
| CD40 | 0.006 | 0.036 | 0.017 | 0.008 | 0.036 | 0.032 | 0.744 | 0.791 |  |
| CD62P | 0.013 | 0.012 | 0.005 | 0.004 | 0.001 | 0.006 | 0.132 | 0.545 |  |
| CD11c | 0.003 | 0.023 | 0.005 | 0.005 | -0.005 | 0.014 | 0.458 | 0.612 |  |
| CD81 | 1.657 | 0.280 | 1.657 | 0.116 | 1.760 | 0.129 | 0.494 | 0.612 |  |
| MCSP | 0.002 | 0.038 | 0.014 | 0.006 | 0.007 | 0.007 | 0.494 | 0.612 |  |
| CD146 | 0.013 | 0.024 | 0.009 | 0.012 | 0.010 | 0.006 | 0.430 | 0.612 |  |
| CD41b | 0.007 | 0.019 | 0.014 | 0.010 | -0.001 | 0.020 | 0.649 | 0.711 |  |
| CD42a | 0.014 | 0.015 | 0.016 | 0.005 | 0.002 | 0.017 | 0.494 | 0.612 |  |
| CD24 | 0.572 | 0.461 | 0.736 | 0.533 | 1.036 | 0.602 | 0.322 | 0.612 |  |
| CD86 | 0.003 | 0.020 | 0.017 | 0.007 | 0.005 | 0.017 | 0.231 | 0.561 |  |
| CD44 | 0.161 | 0.053 | 0.149 | 0.050 | 0.298 | 0.169 | 0.249 | 0.564 |  |
| CD326/EpCAM | 0.694 | 0.668 | 0.283 | 0.153 | 0.229 | 0.134 | 0.176 | 0.545 |  |
| CD133/1 | 0.960 | 1.112 | 0.297 | 0.303 | 0.355 | 0.301 | 0.585 | 0.663 |  |
| CD29 | 0.100 | 0.086 | 0.080 | 0.004 | 0.081 | 0.020 | 0.825 | 0.825 |  |
| CD69 | 0.000 | 0.010 | 0.013 | 0.011 | -0.008 | 0.013 | 0.122 | 0.545 |  |
| CD142 | 0.009 | 0.015 | 0.009 | 0.006 | -0.025 | 0.027 | 0.132 | 0.545 |  |
| CD45 | 0.044 | 0.061 | 0.027 | 0.018 | -0.012 | 0.058 | 0.154 | 0.545 |  |
| CD20 | 0.001 | 0.028 | 0.014 | 0.013 | -0.007 | 0.010 | 0.214 | 0.560 |  |
| CD14 | 0.068 | 0.046 | 0.040 | 0.028 | 0.113 | 0.060 | 0.073 | 0.545 |  |
| CD31 | 0.014 | 0.011 | 0.007 | 0.003 | -0.008 | 0.015 | 0.090 | 0.545 |  |

**Table S6.** Baseline clinical characteristics and corresponding PF-sEV mode size (measured by NTA) for samples used in CD47 co-expression analysis using the MACSPlex EV Kit IO.

| Patient ID | EM staging | Menstrual cycle | Age | BMI | Mode size (nm) |
| --- | --- | --- | --- | --- | --- |
|  | (0-4) |  |  |  |  |
| S1 | 1 | NA | 26 | 22 | 110 |
| S2 | 2 | Irregular | 32 | 33 | 148 |
| S3 | 3 | NA | 31 | 19 | 109 |
| S4 | 4 | NA | 36 | 20 | 250 |
| S5 | 1 | Proliferative | 34 | 23 | 188 |
| S6 | 0 | NA | 29 | 25 | 126 |
| S7 | 0 | Secretory | 22 | 30 | 212 |
| S8 | 0 | NA | 38 | 23 | 247 |
| 1 | 0 | Secretory | 32 | 23 | 130 |
| S9 | 0 | NA | 33 | 27 | 131 |
